# Thermostabilization of VPR, a kinetically stable cold adapted subtilase, via multiple proline substitutions into surface loops

**DOI:** 10.1038/s41598-020-57873-3

**Published:** 2020-01-23

**Authors:** K. R. Óskarsson, A. F. Sævarsson, M. M. Kristjánsson

**Affiliations:** Department of Biochemistry, Science Institute, University of Iceland, Reykjavík, Iceland

**Keywords:** Biochemistry, Biophysical chemistry, Proteases

## Abstract

Protein stability is a widely studied topic, there are still aspects however that need addressing. In this paper we examined the effects of multiple proline substitutions into loop regions of the kinetically stable proteinase K-like serine protease VPR, using the thermostable structural homologue AQUI as a template. Four locations for proline substitutions were chosen to imitate the structure of AQUI. Variants were produced and characterized using differential scanning calorimetry (DSC), circular dichroism (CD), steady state fluorescence, acrylamide fluorescence quenching and thermal inactivation experiments. The final product VPR_ΔC__N3P/I5P/N238P/T265P was greatly stabilized which was achieved without any noticeable detrimental effects to the catalytic efficiency of the enzyme. This stabilization seems to be derived from the conformation restrictive properties of the proline residue in its ability to act as an anchor point and strengthen pre-existing interactions within the protein and allowing for these interactions to prevail when thermal energy is applied to the system. In addition, the results underline the importance of the synergy between distant local protein motions needed to result in stabilizing effects and thus giving an insight into the nature of the stability of VPR, its unfolding landscape and how proline residues can infer kinetic stability onto protein structures.

## Introduction

**Figure 1:**
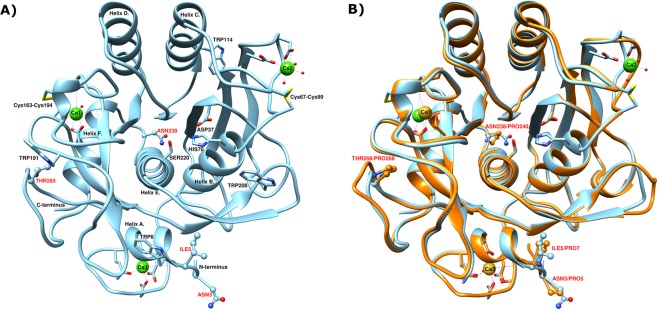
(**A)** The three-dimensional structure of VPR (PDB ID: 1SH7). Residues shown as sticks and balls and marked with red labels are the native residues mutated to prolines in this study. In addition, the catalytic triad Ser220, His70 and Asp37 are also shown along with all the Trp residues in the structure as well as calcium ion coordinators. Calcium ions are shown as green spheres. (**B)** Superimposed three-dimensional structures of VPR (light blue) (PDB ID: 1SH7) and AQUI (Orange) (PDB ID: 4DZT). The native prolines of AQUI are shown as sticks and balls along with the native VPR residues mutated to prolines. Calcium ions are shown as green spheres for VPR and golden spheres for AQUI. Atomic specifiers for side chains are as follows: carbon atoms are coloured same as the secondary structure; nitrogen atoms are coloured blue; oxygen atoms are coloured red and sulphur atoms coloured yellow.

Stabilization of proteins against various environmental factors are of interest in many fields of industry and science, as application range and storage limit their usability in various processes. One of these factors, temperature, plays a pivotal role in this regard. In nature, temperature is one of the main evolutionary drivers of enzymes due to its direct effect on kinetic energies involved in biochemical reactions necessary to maintain life. With early life likely to have existed at high temperatures^1–3^, many branches of life have had to adapt to colder environments over time and thus overcoming slower reaction rates, with no pressure on selecting for thermostable proteins. Many examples of highly active unstable enzymes and thermostable enzymes with low activities at ambient temperatures exist^4–7^. Observations like these prompted the activity/stability trade-off hypothesis. It states that to achieve high stability, molecular motions needed for rapid catalysis at lower temperatures are sacrificed. A recent study has however indicated that adenylate kinases from organisms that throughout their evolutionary history have evolved to cooler temperatures and again toward higher temperatures still retain relatively higher activities at lower temperatures, as in the case of *B. stearothermophilus*^8^. In contrast, thermostable enzymes that have never adapted to lower temperatures showed a much steeper dependence on temperature in order to maintain catalytic rates, as in the case of the enzyme from *C. subterraneus* and *A. aeolicus*^8^. This indicates that the activity/stability trade-off is more of an evolutionary artifact due to different evolutionary pressures, rather than an absolute relationship. In addition, lessons learnt from directed evolution on subtilisins also indicated that the activity/stability trade-off did not share a strict relationship, as higher stability without compromising activity at low temperatures could be achieved^9^ and higher activity at lower temperatures was possible without the loss of stability^10^. Working on that premise, thermostabilization of enzymes from cold adapted organisms would be a feasible method in designing stable enzymes that are highly active at a broad range of temperatures. One of the ways to achieve that goal would be by engineering of cold adapted enzymes via site directed mutagenesis using their thermostable structural homologs as templates. Thus, the aim of this study was to enhance the stability of a kinetically stable, cold adapted subtilisin-like serine protease, VPR^5^ and gain more insight into the molecular basis of kinetic stability of proteins. To this end a truncated version of VPR, VPR_ΔC_^11^, was subjected to single point mutations incorporating the desired proline residues. The positions of proline residues were decided by using structural information from the thermostable structural homologue AQUI^5,12^. AQUI has four proline residues in loops not found in VPR, two of which are located near to the N-terminus, a third is located near a short loop between helices E and F in position 238 (VPR numbering) and the fourth is located on a loop following helix F (position 265) (Fig. 1)^13^. Although many aspects of thermostability have been identified^14^ there is an observation of increased occurrence of proline residues in thermostable proteins^14–17^. This trend seems to be rather prevalent, genomic analysis of five cryophile genomes revealed a trend towards lower proline content in their proteomes^18^. Proline is unique among the natural amino acid residues in protein structures in containing a secondary amine group. This structural fact is the basis of the unique properties of the residue that restrict allowed conformations of the peptide backbone^19^. The effect of these restrictions is of interest with regards to kinetic stability. Kinetically stable proteins unfold irreversibly thus rely on high free energy barriers between the native and denatured states to maintain their activity^20^. Kinetically stable proteases also have evolved to have rigid native states that unfold in a highly cooperative manner^21,22^. Thus, the restriction of movements caused by proline substitutions could enforce pre-existing interaction within the protein structure^23,24^. In this study eight different proline variants were produced, purified and their properties measured. The variants produced were the single proline variants VPR_ΔC__N3P, VPR_ΔC__I5P, VPR_ΔC__N238P, VPR_ΔC__T265P, the double proline variant VPR_ΔC__N3P/I5P, the triple proline variants VPR_ΔC__N3P/I5P/N238P and VPR_ΔC__N3P/I5P/T265P and lastly the quadruple proline variant VPR_ΔC__N3P/I5P/N238P/T265P. The effects of these mutations on the properties of the enzyme were studied by circular dichroism (CD), differential scanning calorimetry (DSC), steady state fluorescence, acrylamide fluorescence quenching and Michaelis-Menten kinetics. The aim of enhancing the stability of VPR_ΔC_ was successful, as the final product, the quadruple proline variant was significantly stabilized without losing catalytic efficiency. In addition, the measured effects of proline exchange of the different variants did shed some light on the mode of action by which proline residues confer stability to the structure of VPR. The observed effects of prolines can be interpreted as restriction of movements leading to strengthening of pre-existing interactions by anchoring certain points within the structure that may lead to more allowed movements within the structure at higher temperatures without unfolding taking place. The effects of some proline substitutions showed clear signs of high local stabilization and as a result unfolding intermediates were observed as cooperativity of unfolding is lost to some degree. However, incorporating proline residues at distant parts of the protein displays synergic effects causing overall higher stability of the protein structure.

## Results

All proline variants were successfully overexpressed in the *E. coli* strain Lemo21 and purified to homogeneity following the reformed production and purification protocol^25^. Thus, all single and the N3P/I5P proline variants^26^ have been recharacterized with respect to activity, thermal inactivation (T_50%_) and the melting of the secondary structure (T_m (CD)_).

### Kinetics

Kinetic parameters determined by Michaelis-Menten assays at 25 °C and pH 8.6 indicated only small changes in turnover numbers and affinity for the substrate for the different variants (Table 1). The only exception was the VPR_ΔC__T265P variant, where k_cat_ and K_m_ were consistently measured a little lower than for VPR_ΔC_ but resulted however in a similar value in terms of catalytic efficiency. The double and both triple proline variants showed little changes in turnover numbers, however this was accompanied by a trend towards slightly higher K_m_ values. The final product of this study, the quadruple proline variant, also still retained its catalytic efficiency as compared to VPR_ΔC_ but had a slightly higher turnover number and K_m_ value.

**Table 1:** Kinetic parameters of VPR_ΔC_ and proline variants. Values are expressed as the averages and the standard deviations of the mean.

Variant	k_cat_ (s^−1^)	K_m_ (mM)	k_cat_/K_M_ (s^−1^mM^−1^)
VPR_∆C_	225.7 ± 12.0	0.177 ± 0.016	1238 ± 149
VPR_∆C_/N3P	235.4 ± 21.8	0.173 ± 0.013	1364 ± 60
VPR_∆C_/I5P	201.6 ± 8.2	0.187 ± 0.010	1077 ± 37
VPR_∆C_/N238P	224.6 ± 16.6	0.189 ± 0.026	1196 ± 84
VPR_∆C_/T265P	166.5 ± 11.6	0.152 ± 0.019	1101 ± 104
VPR_∆C_/N3P/I5P	231.8 ± 10.5	0.187 ± 0.009	1243 ± 77
VPR_∆C_/N3P/I5P/N238P	229.5 ± 18.3	0.199 ± 0.024	1158 ± 72
VPR_∆C_/N3P/I5P/T265P	221.5 ± 7.8	0.219 ± 0.014	1017 ± 74
VPR_∆C_/N3P/I5P/N238P/T265P	259.3 ± 27.4	0.212 ± 0.014	1222 ± 95

### Fluorescence steady state emission and acrylamide quenching

**Figure 2:**
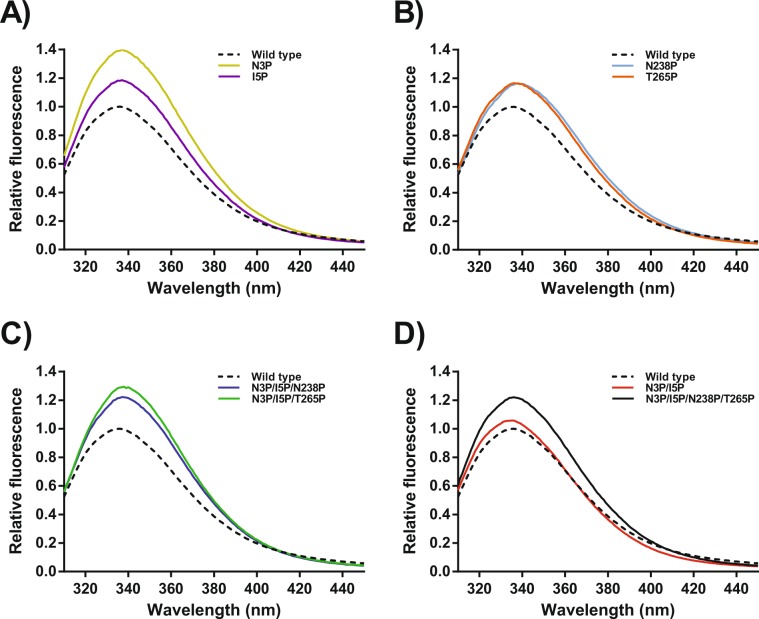
Fluorescence emission of proline variants after excitation at 295 nm at pH 8.0. Intensities of emissions have been normalized against VPR_ΔC_ (dotted black line). (**A)** Emission of VPR_ΔC__N3P (Gold) and VPR_ΔC__I5P (purple). (**B)** Emission of VPR_ΔC__N238P (light blue) and VPR_ΔC__T265P (orange). (**C)** Emission of VPR_ΔC__N3P/I5P/N238P (blue) and VPR_ΔC__N3P/I5P/T265P (green). (**D)** Emission of VPR_ΔC__N3P/I5P (red) and VPR_ΔC__N3P/I5P/N238P/T265P (black).

**Figure 3:**
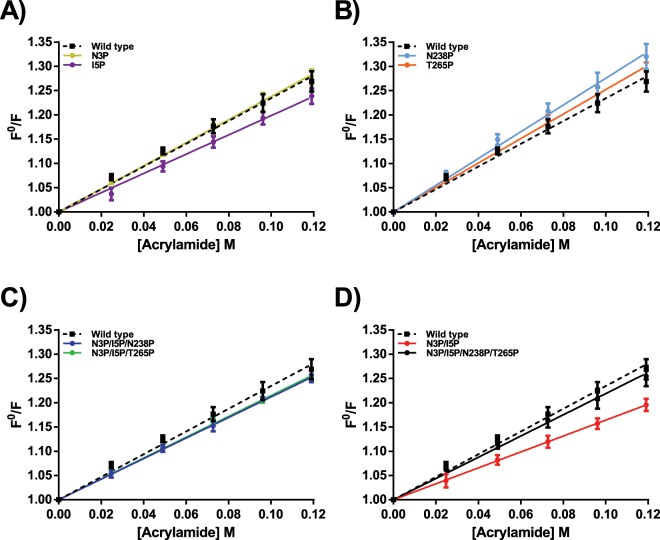
Stern-Volmer graphs calculated form fluorescence quenching of proline variants between 310–410 nm at pH 8.0. VPR_ΔC_ (black boxes with a dotted black line). (**A)** Quenching of VPR_ΔC__N3P (Gold) and VPR_ΔC__I5P (purple). (**B)** Quenching of VPR_ΔC__N238P (light blue) and VPR_ΔC__T265P (orange). (**C)** Quenching of VPR_ΔC__N3P/I5P/N238P (blue) and VPR_ΔC__N3P/I5P/T265P (green). (**D)** Quenching of VPR_ΔC__N3P/I5P (red) and VPR_ΔC__N3P/I5P/N238P/T265P (black).

VPR contains four Trp residues (Trp6, Trp114, Trp191 and Trp208) (Fig. 1). In the native state of VPR these Trp residues are highly intrinsically quenched. This is well demonstrated by examination of the native and denatured steady state fluorescence spectra of VPR_ΔC_ (Supplementary Fig. 1 and Supplementary Table 1) where the denatured state was found to be ten times as fluorescent as the native state at 25 °C. This makes the protein sensitive to changes of the microenvironments around these Trp residues. All single proline variants showed a trend of higher λ_max_ values (Table 2) indicating higher polarity around one or more of the Trp residues. These changes were notably higher for the VPR_ΔC__N238P and VPR_ΔC__T265P variants and these variants were also more quenchable by acrylamide than the wild type and had around 16% higher fluorescence, strongly suggesting changes in the environment of at least one Trp residue. The VPR_ΔC__N3P variant had 37% higher fluorescence than the wild type, but had an unchanged Stern-Volmer constant, but with a minor increase in λ_max_. VPR_ΔC__I5P had a lower Stern-Volmer constant with 16% higher fluorescence and a minor increase in λ_max_. Possibly this indicates some changes in the environment of Trp6 due to its proximity to these mutation sites. The fluorescence properties of the double proline variant VPR_ΔC__N3P/I5P were different from the single variants. VPR_ΔC__N3P/I5P had the same relative amplitude as VPR_ΔC_ but with a slight blue shift in the spectrum indicating a more buried Trp residue. This was further supported by the quenching data as VPR_ΔC__N3P/I5P had a considerably lower Stern-Volmer constant than VPR_ΔC_, indicating reduced flexibility of the N-terminal and/or different dynamics of the N-terminus. These effects of the N3P/I5P mutation seem to be undone by the addition of N238P and T265P, as both relative intensity and the Stern-Volmer constants were higher for the triple proline variants. Those observations however, are likely to be due to changes in accessibility of Trp residues other than Trp6 as these effects are similar as seen by these mutations on the wild type. The quadruple proline variant exhibited similar properties as the triple variants but with a small blue shift in its spectrum (Figs. 2 and 3). The effects of temperature on fluorescence properties were also investigated. Temperatures measured were 15 °C, 25 °C and 35 °C. All variants were stable under those conditions during measurements as seen in the λ_max_ values and the gradual lowering in relative fluorescence intensities (Supplementary Tables 1 and 2). At these temperatures accessibility to fluorophores did not seem so be affected to any extent as Stern-Volmer constants showed just a marginal trend of higher Stern-Volmer constants, often under one standard deviation (Supplementary Table 3). This is consistent with the notion that kinetically stable proteinases are highly rigid structures to reduce auto-proteolysis in their native state^22,27^. However, there is some information to be obtained from these results recorded between 15 °C and 35 °C. Cooperative effects caused by the combination of N238P and T265P on top of the VPR_ΔC__N3P/I5P variant seem to cause the native structure of the final product to become less responsive to acrylamide quenching as a function of temperature, which might be indicative of a more rigid and temperature tolerant structure because of changed dynamics within the protein due to synergy between N238P and T265P.

**Table 2:** Relative intensity of fluorescence calculated as area under the curve (AUC), the maxima of curves (λ_max_) and the Stern-Volmer constant at 25 °C and pH 8.0 of VPR_ΔC_ and the proline variants.

Variant	Stern-Volmer (M^-1^)	λ_max_ (nm)	Relative intensity
VPR_∆C_	2.24 ± 0.12	335 ± 1	1.00 ± 0.03
VPR_∆C_/N3P	2.32 ± 0.18	337 ± 2	1.37 ± 0.02
VPR_∆C_/I5P	2.05 ± 0.13	337 ± 2	1.16 ± 0.05
VPR_∆C_/N238P	2.63 ± 0.25	339 ± 2	1.16 ± 0.07
VPR_∆C_/T265P	2.41 ± 0.14	338 ± 1	1.15 ± 0.08
VPR_∆C_/N3P/I5P	1.64 ± 0.07	334 ± 1	1.00 ± 0.03
VPR_∆C_/N3P/I5P/N238P	2.18 ± 0.05	339 ± 1	1.18 ± 0.08
VPR_∆C_/N3P/I5P/T265P	2.17 ± 0.05	338 ± 1	1.24 ± 0.03
VPR_∆C_/N3P/I5P/N238P/T265P	2.12 ± 0.19	336 ± 2	1.19 ± 0.06

Values are expressed as the averages and the standard deviations of the mean.

### Stability

The impact of proline exchange on the stability of VPR_ΔC_ showed strong evidence for local stabilizing effects. This is best seen in the DSC thermograms with the emergence of an unfolding intermediate. None of the proline variants produced seem to have any notable effects on the secondary structure of the native state as seen in their CD wavelength spectra, the only notable changes being ascribed to concentration estimations i.e. the calculated depth of the spectra (Supplementary Fig. 2). However, structural changes were observed in the microenvironments of Trp fluorophores (Table 2) resulting from these mutations as shown in the steady-state spectra and acrylamide quenching data sets of proline variants (Figs. 2 and 3).

### The N-terminal variants VPR_ΔC__N3P and VPR_ΔC__I5P

**Figure 4:**
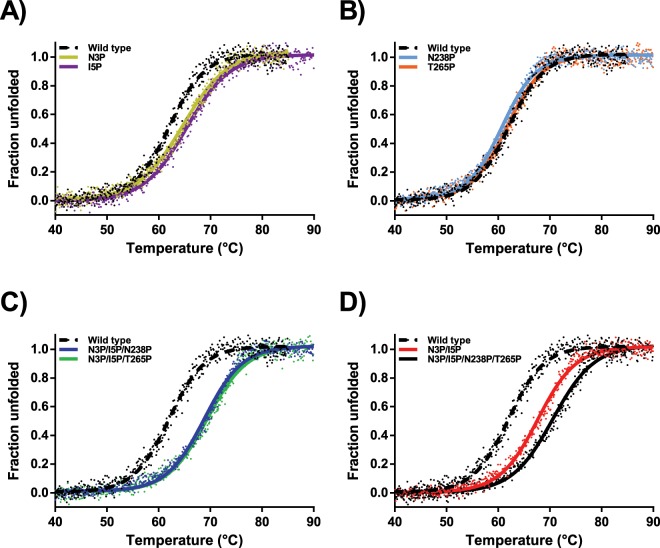
Normalized melting curves of proline variants in glycine buffer containing 15 mM CaCl_2_ and 100 mM NaCl. VPR_ΔC_ (black dotted line). (**A)** Melting of VPR_ΔC__N3P (Gold) and VPR_ΔC__I5P (purple). (**B)** Melting of VPR_ΔC__N238P (light blue) and VPR_ΔC__T265P (orange). (**C)** Melting of VPR_ΔC__N3P/I5P/N238P (blue) and VPR_ΔC__N3P/I5P/T265P (green). (**D)** Melting of VPR_ΔC__N3P/I5P (red) and VPR_ΔC__N3P/I5P/N238P/T265P (black).

**Figure 5:**
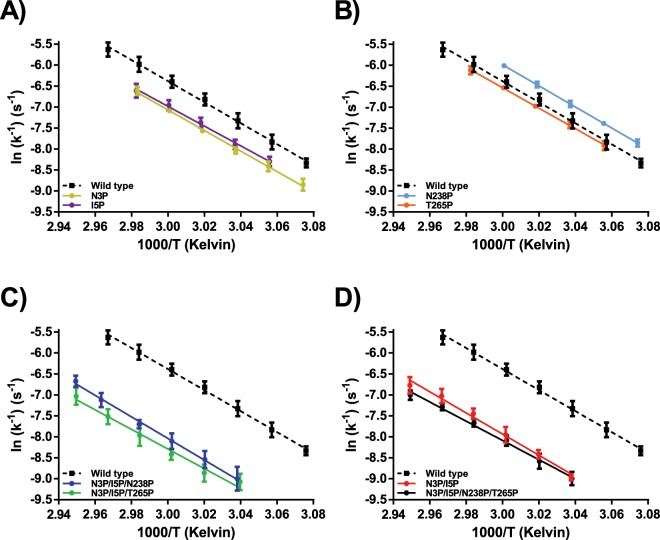
Arrhenius plots calculated form the thermal inactivation of proline variants in Tris buffer containing 15 mM CaCl_2_. VPR_ΔC_ (black boxes with a dotted black line). (**A)** Thermal inactivation of VPR_ΔC__N3P (Gold) and VPR_ΔC__I5P (purple). (**B)** Thermal inactivation of VPR_ΔC__N238P (light blue) and VPR_ΔC__T265P (orange). (**C)** Thermal inactivation of VPR_ΔC__N3P/I5P/N238P (blue) and VPR_ΔC__N3P/I5P/T265P (green). (**D)** Thermal inactivation of VPR_ΔC__N3P/I5P (red) and VPR_ΔC__N3P/I5P/N238P/T265P (black).

**Figure 6:**
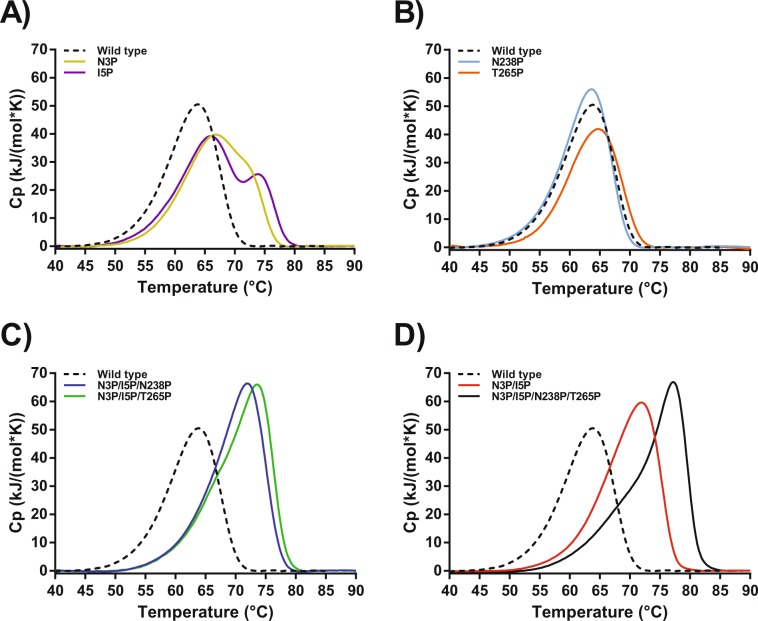
Deconvoluted differential scanning thermograms showing the excess heat during the unfolding process of the proline variants in a glycine buffer containing 15 mM CaCl_2_ and100 mM NaCl. VPR_ΔC_ (dotted black line). (**A)** Unfolding of VPR_ΔC__N3P (Gold) and VPR_ΔC__I5P (purple). (**B)** Unfolding of VPR_ΔC__N238P (light blue) and VPR_ΔC__T265P (orange). (**C)** Unfolding of VPR_ΔC__N3P/I5P/N238P (blue) and VPR_ΔC__N3P/I5P/T265P (green). (**D)** Unfolding of VPR_ΔC__N3P/I5P (red) and VPR_ΔC__N3P/I5P/N238P/T265P (black).

**Figure 7:**
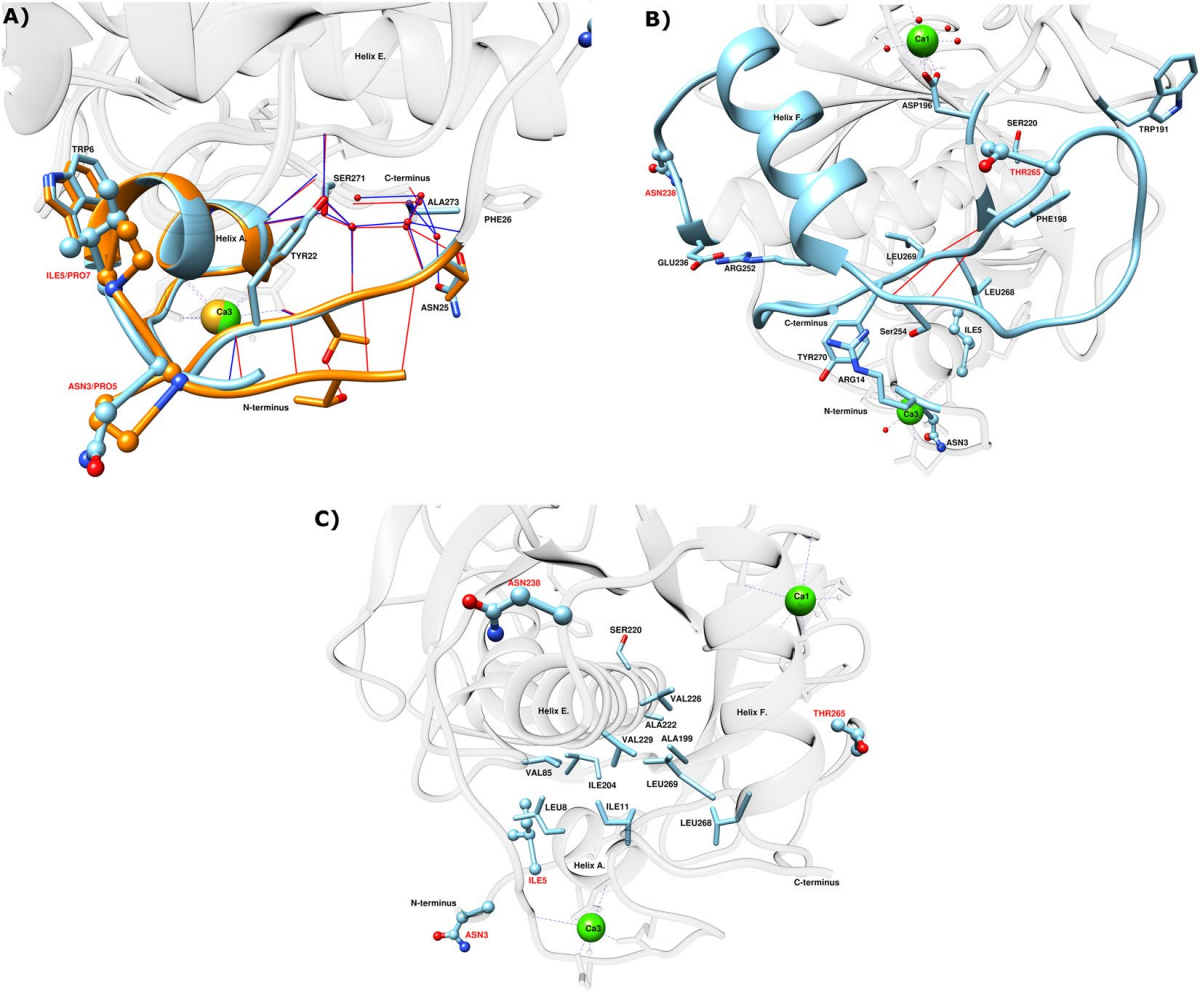
(**A)** Superimposed closeup of the N-terminals of VPR (light blue) (PDB ID: 1SH7) and AQUI (orange) (PDB ID: 4DZT) comparing the H-bond potential of both enzymes (red lines H-bonds in AQUI and blue lines H-bonds in VPR). (**B)** Closeup of the part of the protein in the closest vicinity of the N238P and T265P mutations in the structure of VPR. (**C)** Closeup highlighting the residues making up a part of the hydrophobic core and N-terminal interface to the main part of the protein in the structure of VPR. Calcium ions are shown as green spheres for VPR and golden spheres for AQUI. Atomic specifiers for side chains are as follows: carbon atoms are coloured same as the secondary structure; nitrogen atoms are coloured blue; oxygen atoms are coloured red and sulphur atoms coloured yellow.

Of the single proline variants, the N-terminal mutations had the most impact on the measured melting points as determined by CD and the rates of thermal inactivation (Table 3). VPR_ΔC__N3P and VPR_ΔC__I5P had higher melting points of the secondary structure (T_m (CD)_) by 2.9 °C and 3.2 °C, respectively (Fig. 4) and T_50%_ by 2.8 °C and 2.3 °C (Fig. 5). Accompanied with this increased stability was a considerable change observed in the recorded DSC thermograms of these variants. As for the truncated wild type, the curvature of the thermogram resembles a classic thermogram of a kinetically stable protein that unfolds in a rather cooperative manner following a two-state unfolding model (Fig. 6 and Supplementary Fig. 4)^28^. However, the thermograms of VPR_ΔC__N3P and VPR_ΔC__I5P exhibit a second transition peak, present with a maximum at considerably higher temperature than T_m (CD)_, or around 71.5 °C for VPR_ΔC__N3P (Fig. 6 and Supplementary Fig. 5) and 74.1 °C for VPR_ΔC__I5P (Fig. 6 and Supplementary Fig. 6). This shows that the major part of the three-dimensional structure had dissipated at or around 70– 80% according to CalFitter global fitting of CD melting curves that were recorded at 222 nm in tandem with DSC thermograms (Supplementary Figs. 4 and 5). This might indicate that the local stability of the N-terminus had been increased to such an extent that the cooperativity of the unfolding process was disrupted, thus leading to this apparent intermediate. In the case of VPR_ΔC__N3P the activation energy (E_act_) of unfolding for the first transition was increased when compared to the wild type (Table 4). However, when comparing VPR_ΔC__I5P, where the loss of cooperativity in the unfolding process was even more pronounced, to that of the wild type, the E_act_ values for the first transition did not change but was followed by the second transition having a much higher E_act_. This infers that the first transition state is not as entropically favoured and that a good part of the enthalpic interactions are concentrated within the regions of the protein stabilized by the N-terminus and within the N-terminus itself, as E_act_ reflects on the activation enthalpy of unfolding. The N-terminus, where both these mutations are located also harbours the calcium 3 binding site, which has been suggested to be highly important for the overall stability of the enzyme (Fig. 7)^25^. Combined with the high activation energy of the second transition, this could indicate that the intermediate still retains the calcium-binding site 3. Even though VPR_ΔC_ unfolds in a rather cooperative manner, an elucidation of the chronological order of events during the thermal unfolding of wild type VPR using MD has been reported^29^. There, helix D close to the Ca1 binding site appeared to be the initiation point of thermal unfolding. However, partial unfolding of helix A started soon thereafter and completely dissipated shortly after, but the Ca3 loop seemed to retain itself much longer. In the light of those restricting movements around the N-terminal site of helix A (Fig. 7) as a result of proline substitutions likely slows down the unfolding of that region substantially, promoting the appearance of an unfolding intermediate. According to these unfolding simulations helix E is one of the most stable parts of the protein and accounts for approximately 20% of the helical content of VPR. Thus, speculations that the unfolding intermediate might be consisting of the N-terminal calcium binding loop and helix E fits nicely.

**Table 3:** Thermostability parameters of VPR_ΔC_ and its proline variants.

Variant	T_m (CD)_ (°C)	T_m (DSC)_ (°C)	ΔH_cal_ (kJ/mol)	T_50%_ (°C)	E_act (inactivation)_ (kJ/mol)	t_1/2 (60_ _°C__)_ (min)
VPR_∆C_	61.9 ± 0.4	63.9 ± 0.3	528 ± 35	53.8 ± 0.4	218 ± 9	7 ± 1
VPR_∆C__N3P	64.8 ± 0.1	66.8 ± 0.3	533 ± 33	56.6 ± 0.3	203 ± 12	14 ± 1
VPR_∆C__I5P	65.1 ± 0.2	65.7 ± 0.5	570 ± 11	56.1 ± 0.2	199 ± 14	13 ± 1
VPR_∆C__N238P	60.7 ± 0.1	63.6 ± 0.2	556 ± 8	52.3 ± 0.2	209 ± 17	5 ± 1
VPR_∆C__T265P	61.6 ± 0.2	64.5 ± 0.2	451 ± 51	54.3 ± 0.2	206 ± 4	8 ± 1
VPR_∆C__N3P/I5P	67.8 ± 0.3	72.0 ± 0.8	646 ± 38	60.3 ± 0.4	208 ± 8	33 ± 3
VPR_∆C__N3P/I5P/N238P	68.8 ± 0.2	72.0 ± 0.4	679 ± 37	60.9 ± 0.5	207 ± 27	38 ± 5
VPR_∆C__N3P/I5P/T265P	69.2 ± 0.2	73.6 ± 0.3	697 ± 49	62.2 ± 0.6	194 ± 27	48 ± 7
VPR_∆C__N3P/I5P/N238P/T265P	72.1 ± 0.3	77.2 ± 0.2	683 ± 16	61.6 ± 0.6	182 ± 29	39 ± 3

Parameters shown are T_m (CD)_ the melting point of PMSF inhibited enzymes as measured by CD, T_m (DSC)_ the apparent melting point of PMSF inhibited enzymes defined as the highest peak of DSC thermograms, ΔH_cal_ the excess calorimetric heat released during unfolding, T_50%_ the temperature where half of the activity has been lost over 30 min, E_act (inactivation)_ calculated form the slope of Arrhenius graphs used to calculate T_50%_ and t_1/2_ at 60 °C calculated form the same Arrhenius graphs. Values are expressed as the averages and the standard deviations of the mean.

**Table 4:** Parameters resulting from fitting deconvoluted DSC thermograms (Fig. 6), averaged from at least three separate runs using CalFitter 1.2.

Variant	E_act_^1^ (kJ/mol)	E_act_^2^ (kJ/mol)	ΔH_cal-fit_^1^ (kJ/mol)	ΔH_cal-fit_^2^ (kJ/mol)
VPR_∆C_	235 ± 2	N.A.	542 ± 5	N.A.
VPR_∆C__N3P	251 ± 5	285 ± 20	318 ± 20	227 ± 19
VPR_∆C__I5P	235 ± 3	356 ± 13	399 ± 7	180 ± 6
VPR_∆C__N238P	248 ± 2	N.A.	564 ± 4	N.A.
VPR_∆C__T265P	229 ± 2	N.A.	471 ± 5	N.A.
VPR_∆C__N3P/I5P	261 ± 9	283 ± 6	176 ± 20	477 ± 19
VPR_∆C__N3P/I5P/N238P	270 ± 11	279 ± 3	120 ± 13	567 ± 13
VPR_∆C__N3P/I5P/T265P	259 ± 10	326 ± 8	224 ± 20	481 ± 18
VPR_∆C__N3P/I5P/N238P/T265P	215 ± 8	383 ± 7	275 ± 17	425 ± 15

Values shown are the activation energy (E_act_) of unfolding transitions of PMSF inhibited VPR variants and ΔH_cal-fit_ the calorimetric enthalpy of the fits. Numbers in superscript refer to the chronological order of transitions from the native to unfolded state. All values are represented with their 95% confidence interval.

### The VPR_ΔC__N238P and VPR_ΔC__T265P variants

The VPR_ΔC__N238P variant was the only mutation that caused destabilizing effects, having a ~1.2 °C lower T_m (CD)_ and 1.5 °C lower T_50%_. According to the Arrhenius graphs calculated from the DSC thermograms, VPR_ΔC__N238P (Supplementary Figs. 3 and 7) unfolded cooperatively like the wild type. Even though lowered stability was observed using CD and inactivation experiments, the same observations were not as evident in the DSC thermograms. The apparent melting point (T_m (DSC)_) corresponded very well with that of the wild type, but the variant exhibited higher E_act_ and calorimetric heat of unfolding (ΔH_cal_) (Tables 3 and 4). This suggests that the mutation is indeed enforcing some interactions within the structure, but possibly not as entropically favourable as in the wild type. The VPR_ΔC__T265P variant did cause the smallest changes in the observed stability having T_m (DSC)_, T_m (CD)_ and T_50%_ values around a half a degree higher than that of the wild type. The variant however, had one of the least stable pre and post heat capacities recorded on DSC before and after the unfolding transition, complicating the baseline estimation and data analysis. However, the Arrhenius graph constructed from DSC data (Supplementary Figs. 3 and 8) did indicate some loss of cooperativity during unfolding, but the data could not be reliably fitted to a more complex model than a simple two-state irreversible model, indicating that divergence from linearity observed may have arisen from poor baseline generation.

**Table 5:** Sequences of mutagenic primers used.

Primer	Sequence
N3P fw.	CGTTCAAAGCCCGGCGATTTGGG
N3P rv.	GCTTCATTTGAAACAACAG
I5P fw.	GTTCAAAGCAACGCGCCGTGGGGGCTAGACCG
I5P rv.	CGGTCTAGCCCCCACGGCGCGTTGCTTTGAAC
N3P/I5P fw.	GCCGTGGGGGCTAGACCGAATA
N3P/I5P rv.	GCCGGGCTTTGAACGGCTTCATTTG
N238P fw.	ACAAGAAAACCCGGGCTTAACTCCGCTTC
N238P rv.	AAGTACAAGGCTGCAACG
T265P fw.	AAGAGGCACCCCGAATAAACTGC
T265P rv.	GTGTCAGAAACCTTATTCTC

### The VPR_ΔC__N3P/I5P variant

The double N-terminal proline variant VPR_ΔC__N3P/I5P showed clear additive properties at first glance as it had an increased melting point (T_m (CD)_) by 5.9 °C and T_50%_ by 5.1 °C. However, the DSC thermogram did not exhibit the distinct second peak as was observed for the single proline N-terminal variants. The apparent melting point (T_m (DSC)_) was around 4.2 °C higher than the T_m (CD)_, which is 2.0 °C higher than the difference obtained between T_m (DSC)_ and T_m (CD)_ for the wild type. Also, the T_m (DSC)_ of VPR_ΔC__N3P/I5P more closely coincided with the estimated second peaks from the single N-terminal variants. Moreover, the Arrhenius graph calculated from thermograms showed a slight divergence from linearity and was also most reliably fitted to a three-state model (Supplementary Figs. 3 and 9). This indicates that the unfolding is much more cooperative when the N-terminal proline substitutions are combined in the double variant. This combination of the two most stabilizing proline mutations therefore causes extra stabilization throughout the protein structure, including parts of the protein that did not directly benefit from the single proline mutations. This implies that the single N-terminal proline mutations better stabilize the N-terminus, but synergic effects caused by the combination of N3P and I5P lead to global stabilization of the structure. This effect may be partly explained by a two amino acid residue shift in the auto-cleavage site of the N-terminal during maturation of the protease as was reported on previously^26^. The two-residue extension at the N-terminus potentially adds new hydrogen bonds between the N-terminus and the loop following helix A and anchors these interactions via the movement restricting proline residues. In the crystal structure, residues Tyr22 to Phe26 in that loop can form hydrogen bonds via the facilitation of water molecules to residues Ser271 to Ala273 on the C-terminus of the protein, with mainchain-mainchain distances between residues Asn25 and Leu272 being as low as 7.4 Å (Fig. 7). In addition, the hydrophobic interface of helix A on the N-terminus takes part in the formation of a hydrophobic core along with residues on helix E, helix F, residues Leu268 and Leu269 of the C-terminal region that also form a part of this hydrophobic core. Thus, reduced movements of the N-terminal region which are observed in the fluorescence quenching experiments of this variant (Table 2), may facilitate the increased cooperativity of unfolding compared to the single N-terminal variants, by stabilizing interactions between these parts of the protein at higher temperatures. This is achieved by possible extra H-bonds or enforcement of pre-existing ones reflected in the higher E_act_ values that would indicate that the stabilization is rather enthalpic in nature and explaining the higher ΔH_cal_ observed for VPR_ΔC__N3P/I5P and variants containing that mutational combination.

### The VPR_ΔC__N3P/I5P/N238P variant

Due to the considerable stabilization of VPR_ΔC__N3P/I5P it was of interest to see how the addition of the mutations N238P and T265P would impact the stability. VPR_ΔC__N3P/I5P/N238P increased the T_m (CD)_ by 1.0 °C compared to N3P/I5P but had negligible beneficial effects on T_50%_ (Table 3) This variant had the same apparent melting point T_m (DSC)_ as VPR_ΔC__N3P/I5P indicating some higher degree of cooperativity in the unfolding as is reflected in the Arrhenius graph constructed form DSC thermograms (Supplementary Figs. 3 and 10). However, fitting of data using CalFitter a three-state unfolding process was needed to adequately fit it. The results gave E_act_ values (Table 4) slightly higher than for VPR_ΔC__N3P/I5P for the first transition, which was consistent with the effects observed from the N238P mutation on the wild type. The fact that the second transition was almost unaffected suggests that the N238P mutation does not affect the stability of the N-terminal region or parts of the protein directly impacted by the N-terminal mutations. The increased stability of VPR_ΔC__N3P/I5P/N238P is thus localized to parts of the protein corresponding to the first transition. Thus, placing the initiation point of thermal unfolding around that mutation. MD-simulation at different temperatures have suggested that the initiation point of unfolding is helix D^29^. Helix D is proximal to the Ca1 site, a site that is important for the stability of the structure^25,30,31^. Thus, by restricting movements and enforce interactions to that part of the protein might stabilize it at higher temperatures. Why this mutation had destabilizing effects when added to the wild type but stabilizes the structure when VPR_ΔC__N3P/I5P is the template, is an interesting observation. The site of the N238P mutation is on a loop at the C-end of helix E, a helix that may well be one of the more stable parts of the structure^29^. The mutation does cause loss of H-bond potentials that could be the reason for the detrimental effects observed on the wild type template. However, having stabilized the N-terminal region to a higher degree compared to the rest of the protein the loss of H-bond potential does not show detrimental effects. This may mean that the part of the protein affected by the N238P mutation i.e. helix E, helix F and possibly helix D are reliant on the N-terminal and parts directly stabilized by the proline N-terminal mutations. Thus, the discrepancies in the effects of N238P on VPR_ΔC_ and VPR_ΔC__N3P/I5P can be explained if there is a chronological order to events in the unfolding of VPR and its variants. In the case of VPR_ΔC_ the area affected by N238P unfolds early and immediately thereafter the rest of the protein unfolds. In the case of VPR_ΔC__N238P the proline might anchor that part of the protein and rigidify it, but the parts that this area is being anchored to do not provide the matching interactions needed for increased stability, hence the structure unfolds as in the case of the wild type, possibly with more cooperativity. When the N-terminus has been stabilized by the N3P/I5P mutations the anchoring points within the structure crucial for the stability of the N238P region do stay intact at higher temperatures, thus increasing the stability of the variant and increasing the apparent cooperativity of unfolding compared to VPR_ΔC__N3P/I5P (Fig. 6). This shows that the terminal regions and the major bulk of the protein are finely tuned in the wild type to unfold in a very cooperative manner, strengthening the idea that cooperativity in the unfolding of kinetically stable proteins are under some evolutionary pressures. It also suggests that the chronological order of the unfolding events is an aspect worth looking into regarding the effectiveness of proline substitution.

### The VPR_ΔC__N3P/I5P/T265P variant

VPR_ΔC__N3P/I5P/T265P increased the T_m (CD)_ by 1.4 °C, the T_50%_ by 1.9 °C and T_m (DSC)_ by 1.6 °C when compared to VPR_ΔC__N3P/I5P (Table 3). The thermogram for this variant shows clear signs of an intermediate state and was thus fitted to a three-state model (Supplementary Figs. 3 and 11). The T265P mutation had little to no effect on E_act_ of the first transition indicating that the mutation causes the first transition state to be less entropically favored as the melting point is indeed higher, a similar observation as was made in the case of VPR_ΔC__T265P. However, in this case it was very clear that a second transition was present which yielded a considerably higher E_act_ values for VPR_ΔC__N3P/I5P/T265P than VPR_ΔC__N3P/I5P and an increased apparent melting point. This may suggest considerable synergic interaction between the area affected by T265P and the region of the enzyme affected by N3P/I5P. This rings true as the loop where T265P is located interacts with the Ca3 site, most notably Arg14 and has multiple H-bonding potentials with Arg252, Ser254 and Asp 274 along with the addition of cation-π interaction with Tyr270, possibly yielding the higher activation energy of the second peak. Enforcing these interactions would in turn help maintaining the hydrophobic core of the protein due to the proximity to Leu268 and Leu269 to the mutation site (Fig. 7). In addition, Thr265 lies on an adjacent loop to Asp196 a main coordinator of the calcium ion at the Ca1 site with α-carbon distances in the crystal structure of just 7.0 Å (Fig. 7). Anchoring this part of the protein which harbours many interactions between terminals of the protein, in addition of being in close proximity to the Ca1 site, has the potential of facilitating the correct orientations of these interactions at higher temperatures thus explaining the increased stability of both transitions.

### The VPR_ΔC__N3P/I5P/N238P/T265P variant

The final product, VPR_ΔC__N3P/I5P/N238P/T265P was also the most stable variant with a melting point of 72.1 °C (T_m (CD)_), 10.2 °C higher than the wild type and 4.3 °C higher than VPR_ΔC__N3P/I5P. T_50%_ values were however least affected, being slightly lower than that of VPR_ΔC__N3P/I5P/T265P, but yielded an increase of 7.8 °C as compared to the wild type. The largest increase in stability was recorded by DSC with an apparent melting of 77.2 °C (Table 3), which was 13.3 °C higher than the wild type and 5.2 °C higher than VPR_ΔC__N3P/I5P. However, this was accompanied by a very clear unfolding intermediate, with both transitions being more stable than seen in any other variant especially the second transition (Supplementary Fig. 12). A notable change that was observed was the low E_act_ fitted to the first transition and the very high E_act_ of the second transition. This is a similar observation as for VPR_ΔC__I5P, where the overlap of unfolding events was low and the estimated E_act_ of the first transition was also low. This supports the idea that the first part to unfold is a major part of the α/β structure, as its unfolding would lead to the exposure of hydrophobic residues which would be entropically more unfavourable than the subsequent unfolding of the terminal region, along with at least calcium binding site 3. This latter process would be entropically favourable due to the release of the bound calcium ion and as a consequence would transform a rigid calcium binding loop into a flexible loop thus increasing the entropy of the system^32,33^. The synergic effects in this variant show that both transitions are highly stabilized, but the second transition is the more affected. From the VPR_ΔC__N3P/I5P/T265P variant it is clear, that the T265P mutation has synergic effects with the terminal region of the protein. The quadruple proline variant adds implication of synergic effects between N238P and T265P. These two mutation sites are located on loops on the either side of helix F and neighbouring residue of the N238P site is Glu236 on helix E that likely forms a salt-bridge to Arg252 located on helix F. Ser254 located on the loop at the C-end of helix F can form a H-bond to Arg14 as mentioned earlier and is also involved in two mainchain-mainchain H-bonds that can be formed between Ser254 and Leu268, a partner in the hydrophobic core of the protein. In addition, Leu268 forms two mainchain-mainchain H-bonds to Phe198, possibly providing extra stabilization to the Ca1 binding site by restricting the neighbouring Asp196, a main coordinator in that calcium binding site (Fig. 7). Restricting movements at this site might be crucial for maintaining local interactions at higher temperatures, increasing the thermostability of the enzyme resulting from the combined effects of N238P and T265P.

## Discussion

This work focused on VPR_ΔC_, a cold active subtilisin-like serine protease from the proteinase K family^5^. VPR_ΔC_ is expressed as a preproenzyme, containing a N-terminal intramolecular chaperone that is cleaved off during maturation leaving a 28 kDa active protease with an α/β-fold^11,13,34^. This maturation leaves a kinetically stable enzyme in a process that may be similar to what has been described for α-lytic protease^11,13,25,34,35^. During this maturation the calcium binding site 3 (Ca3) (Fig. 1) is likely formed. VPR_ΔC_ contains three calcium binding sites and calcium binding is one of the most important structural factors contributing to the stability of the enzyme. The most important calcium binding sites for the stability are believed to be calcium binding site 1 (Ca1) (Fig. 1), mainly coordinated by the conserved Asp196 residue and Ca3 located in the N-terminal region^25,30^. The third calcium binding site is the low affinity Ca2 site thought to mainly serve as a defence against exogenous proteolysis^25^. The aim of the study was to explore the effects of insertion of proline residues into loops on the stability and activity of VPR and to construct a more stable VPR variant. The aim of creating a more thermostable variant of VPR by proline substitutions into loops was successful and this stabilization was achieved without losing catalytic efficiency. The use of the thermostable structural homolog AQUI as a template for selecting proline mutations was therefore a successful strategy. Research has been carried out on AQUI^36^ where the same proline residues were exchanged to the corresponding residues in VPR, also indicated that the N-terminal proline residues were the most important with regards to thermostability. In that study the DSC thermograms were recorded at pH 7.4 in a filtered phosphate buffer containing 1 mM calcium, that would leave a very low concentration of calcium in the samples after dialysis and filtration^37^. There the mutant AQUI_P7I (corresponding to I5P) exhibited a thermogram with an apparent melting point almost 20 °C lower than the wild type. However, the thermogram of AQUI_P5N (corresponding N3P) a small peak was observed in front of the main transition, which had a similar unfolding initial temperature as AQUI_P7I. In the light of the results obtained for the VPR proline variants in the present study, these rather drastic but varied effects observed in the DSC thermograms of these N-terminal AQUI variants, further support the hypothesis that the Ca3 site is one of more important sites for the stability of these protein structures. The extremely low calcium concentrations in those AQUI experiments may however further exacerbate the destabilizing effects of the N-terminal mutations, as the two corresponding calcium binding sites in AQUI (Ca1 and Ca3) may be partly or fully depleted of calcium due to phosphate coprecipitation. In addition, P240N (corresponding to N238P) and P268T (corresponding to T265P) did also cause destabilization although not to the same extent as in the N-terminal variants. Thus, all these proline residues do serve a stabilizing role in the structure of AQUI.

This study also sheds some light on the way proline residues affect protein kinetic stability. Proline exchanges have been observed to have either a beneficial or detrimental effect on protein stability, even detrimental when mutation sites were selected on basis of structural comparisons to more stable structural homologues^38–41^. What causes these discrepancies in observations made on proline substitutions is not clear and a subject of debate regarding the importance of proline residues in protein structures. In the present study none of the variants showed any significant changes in their secondary structures, according to the far UV CD wavelength scans (Supplementary Fig. 2). An aspect of proline exchanges to consider is the loss of H-bonds due to the cyclic nature of the side chain of the residue which cannot therefore act as a H-bond donor. The N-terminal proline substitutions are not expected to cause any loss of H-bonds based on the crystal structure. The N238P and T265P substitutions however, do cause a loss of hydrogen bonding potential. In the case of N238P the potential of the N238 side-chain to Gln235 main-chain H-bond is lost, possibly, to a degree explaining the loss of stability resulting from this mutation when the wild type was used as a template. In the case of the T265P mutation the most likely H-bond potential to be lost is from the side-chain of Thr265 and the side-chain of Lys267, which effect is expected to be rather benign as it is solvent exposed, thus reducing its expected lifetime in the structure. The only measurable evidence for structural changes in the native state of proline variants were obtained from steady-state fluorescence spectroscopy and acrylamide quenching experiments (Figs. 2 and 3). Quenching experiments showed restricted accessibility to Trp residues accompanied with VPR_ΔC__I5P, and even more in the case of the VPR_ΔC__N3P/I5P variant. In those experiments Trp6 could be acting as a reporter on dynamics of the N-terminal region. The N-terminal mutations are thus likely to be causing restrictions of movements within the N-terminal region. These observations agree with the idea that the disproportional local stabilization within the N-terminus of the protein is caused by the restricted movements resulting from the N-terminal proline residues. This increased local stability leads to the emergence of an unfolding intermediate that could be observed in DSC thermograms of all proline variants containing N-terminal mutations, implicating the N-terminus as a part of the unfolding intermediate. The highly energetic second transition also fits well with observations on the effects of calcium on the calorimetric enthalpy of unfolding for VPR, as it has been shown that the calorimetric enthalpy of denaturation increases with increased calcium concentration in the buffers^25^. Measured melting points by CD do not increase above 10 mM calcium, but the rates of inactivation, the calorimetric enthalpy and the apparent melting point measured by DSC still increase up to 100 mM due to binding to the low affinity Ca2 site (Fig. 1) and the moderate affinity Ca1 site^25^. This shows that in the unfolding of VPR the calcium binding sites contribute greatly to the calorimetric heat evolved during unfolding and that changes to these sites would likely be well observable in DSC thermograms. Stabilization of the overall structure is also achieved however, thus the local stabilization of the N-terminal part reverberates throughout the structure. The N-terminal region interacts with several parts of the protein. Helix A that holds two of the Ca3 coordinating residues also is part of the hydrophobic interface between the N-terminus and the main body of the protein through Ile11 and Leu8 (Fig. 7). The Ca3 binding loop also contains Arg10 that can form a H-bond network with several residues at the C-terminal part of the protein, in addition to cation-π interaction with Tyr270. For VPR_ΔC__N3P/I5P it is apparent that the stability of the second transition of the DSC thermograms is not affected to a large extent but most of the stabilization is reflected in the first transition. VPR_ΔC__N3P/I5P shifts the autocatalytic site by two residues^26^ and this provides more H-bond potential between these extra residues and the loop following helix A in a location where the loop is proximal to the C-terminal parts (Fig. 7). Thus, the N-terminal prolines might be seen acting as anchors maintaining these interactions between distant parts of the protein at higher temperatures. In addition, the stabilization of the protein structure caused by the VPR_ΔC__N3P/I5P creates an environment where N238P and T265P can contribute more to stabilization. Fluorescence quenching experiments of the single N238P and T265P variants indicate changes in the microenvironments of a Trp residue or residues. As seen in lower intrinsic quenching, higher sensitivity to acrylamide quenching and a red shift in λ_max_ indicating a more polar environment. These same observations are very apparent when these mutations are added onto the VPR_ΔC__N3P/I5P template (Table 2). How T265P and N238P effect VPR_ΔC_ and VPR_ΔC__N3P/I5P show that the microenvironment of a Trp residue, likely other than Trp6, is clearly influenced. Considering proximity in the structure, Trp191 is a strong candidate for being that Trp residue, a residue that might probe for change in movements around the Ca1 site and its main coordinator Asp196. Synergic effects between the N238P and T265P mutations indicate reduced movements as a function of temperature on the affected fluorophore as the change in Stern-Volmer constants between 15 °C and 35 °C are lower for VPR_ΔC__N3P/I5P/N238P/T265P compared to the temperature effects observed for VPR_ΔC__N3P/I5P/N238P and VPR_ΔC__N3P/I5P/T265P. These synergic effects observed in fluorescence quenching might indicate that N238P and T265P are indeed affecting the same fluorophore.

In this study we demonstrated that insertion of proline residues into loops contributed significantly to the stability of VPR_ΔC_ variants. The mode of action is likely by restricting movements at critical points in the structure that enforces pre-existing interactions by anchoring certain parts of the protein in correct positions at higher temperatures. The restrictive nature of proline residues could thus decrease the flexibility of the structure at low temperatures but allow for more movement at higher temperatures without losing the structural integrity of the protein by retaining the interactions as more thermal energy is applied to the system. The conclusion of these observations would be that the role of proline residues in loops in the kinetic stability of proteins is to allow for more thermal flexibility of the structure^42^. Proline substitution is an effective way to stabilize kinetically stable proteins, however as shown their position within the protein structure is of utmost importance. Surface loops are good targets due to the structural nature of the residue that may leave some H-bonds unfulfilled within the protein core, possibly causing destabilization of the structure^43,44^. As proline residues seem to enforce important interactions, including interactions between distant parts of the primary structure, it would mean that the effects of prolines in the structure could be rendered useless if a crucial counterpart to the interaction being strengthened is destabilized to a certain degree or not present. Explaining the non-additive nature of combining proline mutations in this study and some of the discrepancies observed in proline mutagenic studies so far. Although the stability of the final product is greatly increased compared to the wild type, an unfolding intermediate was observed in the unfolding process. This may explain the higher degree of stabilization observed in CD and DSC as compared to T_50%_ values and the trend of lower activation energies of inactivation (E_act (inactivation)_) (Table 4) of the more stable proline variants as the intermediate state may be a good target for exogenous proteolysis^27^. Under our standard experimental conditions this intermediate is not observable in the unfolding process of the wild type. However, when unfolding takes place at pH 5 and 1 mM CaCl_2_, conditions known to be destabilizing for the enzyme, an intermediate could be observed (Supplementary Fig. 13). This intermediate bears many similarities to what was observed in case of the VPR_ΔC__I5P unfolding process (Supplementary Figs. 6, 13, 17 and 18). Under these destabilising conditions it may be that calcium binding is compromised. However, if the calcium ion concentration is increased to 15 mM the first transition is stabilized and the intermediate is not as readily observed (Supplementary Fig. 13). These observations fit our ideas concerning the unfolding pathway and the role of the Ca1 binding site^25^, as a compromised Ca1 binding site could lead to destabilisation of a possible unfolding initiation point at helix D^29^. In addition, low pH values could destabilise further interactions important for the stability of the protein structure but less so for the intermediate state. This information thus indicates that there is a metastable intermediate along the unfolding pathway that is poorly structured and which by tweaking conditions, or by increasing the local stability of the N-terminal region can become kinetically trapped along the unfolding pathway causing the apparent loss of cooperativity in the unfolding process. This also suggests that the quadruple variant has the potential to be stabilized even further by tweaking the stability of the first transition and increasing the cooperativity of unfolding. As a result, VPR_ΔC__N3P/I5P/N238P/T265P is a prime candidate for further work exploring enzyme kinetic stability, thermostability, protein engineering and temperature adaptation.

## Materials and Methods

### Site directed mutagenesis

All mutations were done on the gene of VPR_ΔC_ (a C-terminal truncated form of wild type VPR)^13^, contained in a pET-11a-d vector^25^. Proline variants were obtained with site-directed mutagenesis using Q5 site-directed mutagenesis kit from New England Biolabs (NEB) following their protocol. Mutagenic primers used to produce the variants were designed using the web tool NEBaseChanger (NEBaseChanger.neb.com), except for the I5P variant which was made using the Quickchange Site directed Mutagenesis Kit from Stratagene, following their protocol (Table 5). Mutagenic PCR products were all transformed into XL10-Gold from Agilent Technologies, genotype: *TetrD(mcrA)183 D(mcrCB-hsdSMR-mrr)173 endA1 supE44 thi-1 recA1 gyrA96 relA1 lac Hte [F´ proAB lacIqZDM15 Tn10 (Tetr) Amy Camr]*. Plasmid purification was done with the Monarch plasmid miniprep kit from NEB, following their instructions. All mutations were verified by Sanger sequencing performed by Genewiz.

### Expression and purification

All proline variants were expressed in the *E. coli* strain Lemo21 (NEB) from a pET-11a-d vector, utilizing the T7 polymerase/T7 lysozyme system^45^. Liquid media used for the expression of all variants was 2xYT broth containing 0.1 mg/mL ampicillin (Sigma), 0.03 mg/mL chloramphenicol (Sigma) and 76 µM rhamnose (Sigma). Cultures were grown to a density of A_600_ ~ 0.4–0.8 A.U. and expression was initialized by adding isopropyl β-D-1-thiogalactopyranoside (IPTG) (AppliChem) to a final concentration of 400 µM followed by the addition of sterile 4 M CaCl_2_ (Sigma) to give a final concentration of 100 mM and grown at 18 °C and 230 rpm for 20–24 hours. All proline variants were purified to homogeneity as described in^25^.

### Activity assays

All activity assays were performed in 100 mM Tris, 10 mM CaCl_2_ at pH 8.6 using Suc-AAPF-NH-Np as a substrate. Kinetic parameters of proline variants were characterized by Michaelis-Menten assay monitoring activity at 25 °C against Suc-AAPF-NH-Np at seven different substrate concentrations, up to 1.00 mM, and monitoring ΔA_410_ over 15 seconds. Enzyme samples were dialyzed against the assay buffer overnight at 4 °C and concentration was estimated by A_280_ measurements using the calculated molar attenuation coefficient 34,170 M^−1^cm^−1 ^^46^. Exact substrate concentrations were determined at 410 nm, using the molar attenuation coefficient 8,480 M^−1^cm^−1 ^^47^. Data points were then fitted to the Michaelis-Menten equation using the analysis software KaleidaGraph (Synergy Software).

### Fluorescence

Steady state fluorescence was recorded for each variant at 15 °C, 25 °C and 35 °C on a Fluoromax-4 spectrofluorometer (Horiba Scientific) equipped with a circulating water bath for temperature control. All samples were inhibited by PMSF to a final concentration of 2.5 mM followed by dialysis against 50 mM Tris, 10 mM CaCl_2_ and pH 8.0 overnight at 4 °C. Prior to fluorescence experiments absorbance spectra were recorded from 400 nm down to 220 nm and absorbance tuned to 0.03–0.05 A.U. at 295 nm in a 0.4 cm quartz cuvette (Spectrocell) used for fluorescence experiments. In addition to recording native fluorescence of all variants, steady state fluorescence of the denatured state was also recorded for VPR_ΔC_, VPR_ΔC__N3P/I5P and VPR_ΔC__N3P/I5P/N238P/T265P, where samples were heated to 90 °C for 15 minutes and fluorescence measured at 25 °C. All samples were excited at 295 nm using 3 nm entrance slit width and fluorescence monitored between 310 nm and 450 nm using a 5–8 nm exit slit width for native samples and 2–3 nm for denatured samples. Relative fluorescence was then calculated as:

$${F}_{n.}=\frac{\left(\frac{CPS}{[P]\ast exi{.}^{2}}\right)}{{F}_{{\rm{VPR}}\Delta {\rm{C}}}}$$

where F_n._ is the normalized fluorescence intensity, CPS the recorded fluorescence intensity, [P] the protein concentration, exi. the exit slit width used and F_VPRΔC_ the concentration and exit slit width normalized fluorescence for native VPR_ΔC_. The peak of each fluorescence spectra was then fitted to a cubic function, solving the first derivative for the local maximum (λ_max_). AUC (area under curve) was calculated for all variants via the trapezoidal rule and the relative emission efficacy calculated by dividing the results with the average fluorescence intensity for native VPR_ΔC_. In addition, acrylamide quenching was conducted on all variants, using a 2.5 M stock of molecular biology grade acrylamide (Sigma). Sample preparation and experimental conditions were as described above. Each aliquot of acrylamide added to samples was followed by thorough mixing and one min resting time for temperature equilibration. The effectiveness of quenching was calculated by fitting the data with the Stern-Volmer equation:

$$\frac{{{\rm{F}}}^{{\rm{o}}}}{F}=1+{K}_{SV}[Q]$$

where F^0^ and F are the fluorescence intensities in the absence and presence of quencher between 310 nm and 410 nm, [Q] is concentration of quencher and K_sv_ is the Stern-Volmer constant calculated via linear regression. Corrections of fluorescence intensities were performed on the data to account for dilutions due to additions of acrylamide.

### Thermal stability

Prior to thermal inactivation experiments, samples were dialyzed against a 25 mM Tris buffer containing 15 mM CaCl_2_, 100 mM NaCl, 1 mM EDTA and at pH 8.95 (Sigma) overnight at 4 °C. Samples were then heated to selected temperatures and aliquots withdrawn at timed intervals for assaying remaining activity using 0.5 mM Suc-AAPF-NH-Np. The observed first order rate constants were then used to construct Arrhenius-plots that were analysed by linear regression using KaleidaGraph, from which the T_50%_ (the temperature where half of the activity was lost after thirty minutes) and E_act (inactivation)_ (corresponding to the slope of the Arrhenius graph) values were then calculated.

Unfolding of the secondary structure was monitored by circular dichroism (CD). Prior to measurements samples were inhibited by PMSF at a final concentration of 2.5 mM followed by dialysis against a 25 mM glycine buffer containing 100 mM NaCl and 15 mM CaCl_2_ at pH 8.6 overnight at 4 °C. Melting curves of protein samples (0.1–0.4 mg/mL) were recorded at 222 nm with a heating rate of 1 °C/min from 25 °C to 90 °C on a Jasco J-1100 spectropolarimeter. Data analysis and T_m (CD)_ determination was performed as described in^25^. Concurrent CD wavelength scans were also recorded on a Jasco J-1100 from 250 nm down to 200 nm at 25 °C using a 1 mm cuvette.

Differential scanning calorimetry (DSC) was used to record thermograms of the unfolding process using a MicroCal VP-DSC. Prior to measurements, samples were inhibited by PMSF at a final concentration of 2.5 mM followed by dialysis against a 25 mM glycine buffer containing 100 mM NaCl and 15 mM CaCl_2_ and pH 8.6 overnight at 4 °C. Prior to loading, protein samples (0.4–1.2 mg/mL) and buffers were degassed for 15–30 min at 10 °C. Thermograms were then recorded from 15 °C to 95 °C with a temperature gradient of 1 °C/min. Initial data analysis was performed by Origin software where buffer subtraction and concentration normalization was carried out. Due to a slow downward sloping post heat capacities recorded at high temperatures for some variants (Supplementary Fig. 14), the Origin software was used to normalize data sets by baseline generation to convert data sets into plots of excess heat capacity versus temperature. Initial data analysis consisted of calculating the AUC (area under curve) of each excess heat capacity plot via the trapezoidal rule yielding the calorimetric enthalpy (ΔH_cal_), including recalculations for VPR_ΔC_ data sets from^25^. The apparent melting points (T_m (DSC)_) were found by fitting a cubic function to the highest peak of the thermograms and solving the first derivative for the local maximum. The rate of unfolding (k_(unfold)_) was calculated as:

$${k}_{(unfold)}=\frac{v{C}_{p}}{{Q}_{t}-Q}$$

where v is the speed of the temperature gradient, C_p_ is the excess heat capacity at a given temperature, Q_t_ is the total heat evolved and Q is heat evolved at a given temperature^4,25,28,48^. The unfolding rates were then used to plot Arrhenius graphs and used as assistance in further analysis. Due to the complexity of some thermograms CalFitter v1.2 (https://loschmidt.chemi.muni.cz/calfitter/)^49^ was utilized to separate unfolding events. The model CalFitter used for fitting irreversible transitions is a modification of the Arrhenius equation:

$$k=\exp \left(-\frac{{E}_{act}}{R}\left(\frac{1}{T}-\frac{1}{{T}_{act}}\right)\right)$$

where k is the rate of unfolding, E_act_ is the activation energy of unfolding, R is the gas constant, T is the absolute temperature and T_act_ is an expression of the preexponential factor A, that has been transformed into exponent with the single new parameter T_act_ for more robust parameter estimation. The average of normalized DSC scans of variants and a normalized CD melting curves were simultaneously subjected to global fitting using CalFitter. As CalFitter cannot account for the downward slopes as observed in some thermograms the normalized excess heat thermograms were fitted instead. For fitting of normalized curves ΔC_p_ of each transition was fixed at 0 along with the slope (see supplementary for reflections on data analysis). The unfolding model selected for the wild type and variants that did not exhibit complex unfolding was a two-state irreversible model confirmed by DSC scan-rate experiments (Supplementary Fig. 15)^28^. Variants that did exhibit more complex unfolding a three-state model with both transitions being irreversible, was used. The model was chosen by running scan-rate experiments on VPR_ΔC__I5P (the variant with the most prominent second unfolding transition) (Supplementary Figs. 16, 17 and 18) revealing that both transitions exhibited scan-rate independent activation energies with more accumulation of the intermediate at slower scan-rates. In addition, reheating experiments^50^ on VPR_ΔC__I5P up to 70 °C and VPR_ΔC__N3P/I5P/N238P/T265P up to 72 °C showed no signs of refolding (Supplementary Figs. 19 and 20) in case of the first transition. In addition, protein stability dependence on protein concentration which was measured by recording melting points on CD at 0.1 mg/mL, 0.2 mg/mL and 0.4 mg/mL for both VPR_ΔC_ and VPR_ΔC__I5P (Supplementary Fig. 21), showed no concentration dependence for either variant, excluding the possibility of oligomerization causing the observed second transitions.

### Molecular modelling and graphical data representation

Molecular graphics and H-bond analysis was performed with UCSF Chimera^51^ using the crystal structure of AQUI (4DZT) and VPR (1SH7)^13^. Data sets were plotted using GraphPad Prism 6 for Windows, GraphPad software.

## Supplementary information

Supplementary Information.
