# Supplementary material for: Thermostabilization of VPR, a kinetically stable cold adapted subtilase, via multiple proline substitutions into surface loops
Source: Sci Rep. 2020 Jan 23;10:1045. doi: 10.1038/s41598-020-57873-3 (PMC6978356; doi:10.1038/s41598-020-57873-3)
Supplement: Supplementary file 1 — Supplementary Information. [file 41598_2020_57873_MOESM1_ESM.pdf]

Thermostabilization of VPR, a kinetically stable cold adapted subtilase, via multiple proline substitutions into surface loops.

Supplementary.

K. R. Óskarsson, A. F. Sævarsson & M. M. Kristjánsson*

Department of Biochemistry, Science Institute, University of Iceland, Reykjavík, Iceland. Correspondence and requests for materials should be addressed to M.M.K. (email: mmk@hi.is)

Reflections on data analysis.

In this paper we utilized DSC and CD to analyse the unfolding processes of $VPR_{\Delta C}$ and its proline variants. The unfolding process of $VPR_{\Delta C}$ follows a classical two state irreversible pathway as seen from its DSC thermogram (Fig. 6) and resulting Arrhenius graph (Supplementary Fig. 3). However, six out of nine enzyme constructs measured in this work did show evidence of divergences from the cooperative unfolding observed for the wild type. In this paper we utilized CalFitter 1.2 [1] in order to analyse all data sets in the same working environment. However due to unstable pre and post heat capacities of some variants, most notably $VPR_{\Delta C}$_T265P, all data sets were converted into excess heat thermograms. Thus, the change in heat capacity due to unfolding could not be considered. Thus, we opted to report on E_{act} values instead of activation Gibbs free energies, activation enthalpy or activation entropy. Although baseline subtraction leads to a loss of information and possibly precision in parameter estimation, our observation was that the accuracy holds and was improved when working with data sets that showed signs of slow aggregation appearing as downward sloping post heat capacities after the thermogram peak. In addition, proteases such as αLP and SGPB that are produced with intramolecular chaperones and are entirely dependent on them for folding, as is the case for VPR, have shown to have very large $\Delta C_p^{\ddagger}$ and $\Delta G^{\ddagger}$ values [2]. Together this results in highly temperature dependent $\Delta G^{\ddagger}$ curves, making free energy comparisons hard between variants without having a value for $\Delta C_p^{\ddagger}$. Thus, we feel that reporting E_{act} values we get more reliable results for comparison between proline variants as baseline subtraction was always performed in the same manner. For the wild type and variants showing cooperative unfolding the simplest model for irreversible unfolding holds true:

$$N \xrightarrow{k_1} D$$

where N stands for the native state, D for the denatured one and k_1 is the rate of unfolding at a given temperature. Variants that exhibited divergences from the irreversible two-state model

did all fit best to a three-state irreversible model rather than a Lumry-Eyring unfolding. The model that best fitted the data had both steps being irreversible:

$$N \overset{k_1}{\rightarrow} I \overset{k_2}{\rightarrow} D$$

where I stands for the intermediate and k_2 stands for the unfolding rate of the intermediate state at a given temperature and used to calculate the activation energy of the second transition. Partial unfolding experiments also indicated that the first transition was irreversible or at least slow enough that it did not come into effect during experiments (Supplementary Fig. 19 & 20).

Supplementary Figures and Tables.

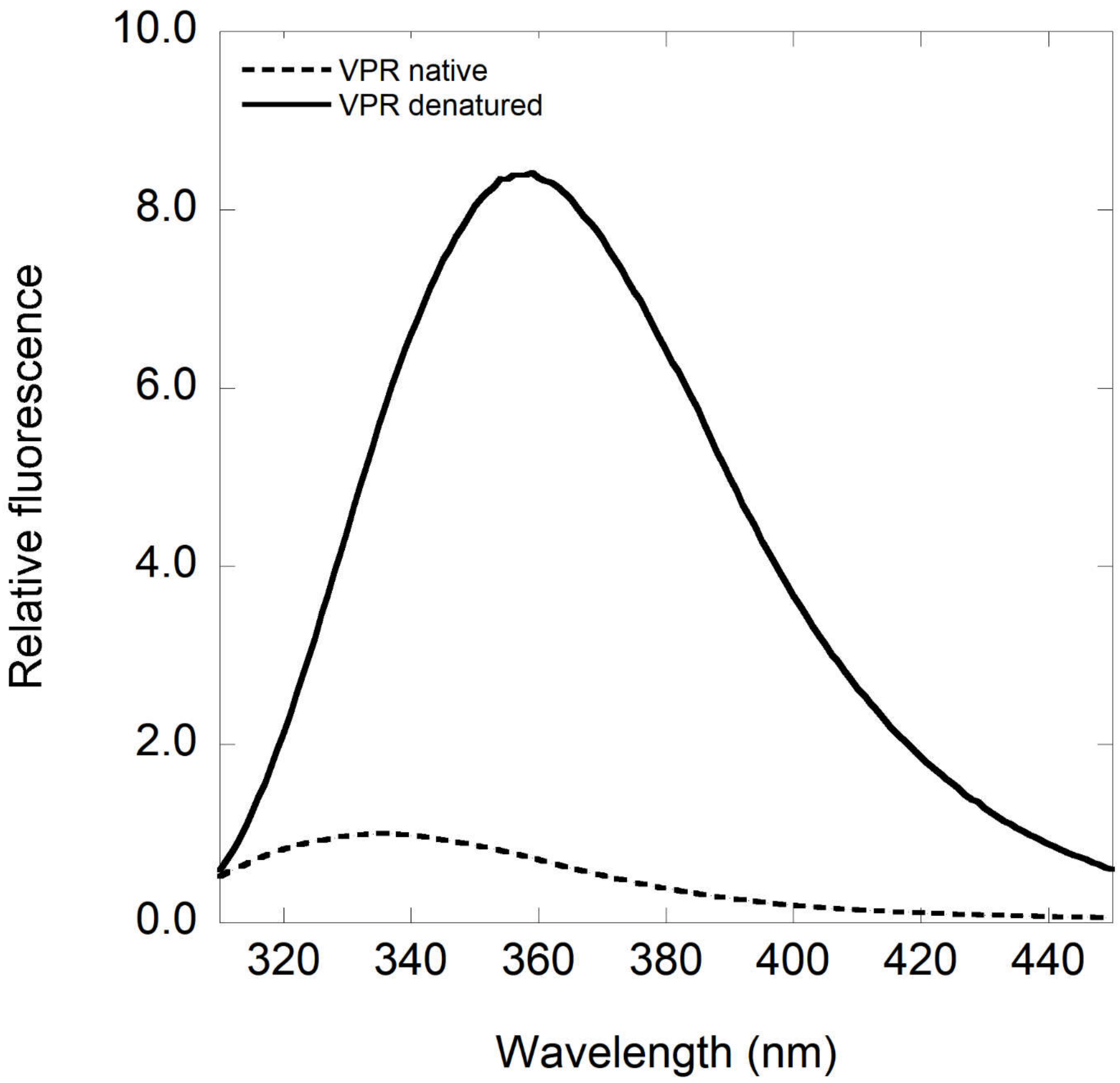

Supplementary Fig. 1. Fluorescence emission of $VPR_{\Delta C}$ at 25°C and pH 8.0 before heat treatment (dotted line) and after heat treatment (solid line). Heat treatment was carried out by heating at 85 °C for 15 min and then cooled down before measurements.

Supplementary Table 1. Relative fluorescence intensities of proline variants calculated as the AUC of fluorescence curves at different temperatures and of the denatured state at 25 °C. Along with the standard deviation of the mean.

Variant	15 °C (Relative intensity)	25 °C (Relative intensity)	35 °C (Relative intensity)	Denatured 25 °C (Relative intensity)
**VPR$_{\Delta C}$**	1.12 ± 0.02	1.00 ± 0.03	0.95 ± 0.03	10.02 ± 0.04
**VPR$_{\Delta C}$/N3P**	1.46 ± 0.08	1.37 ± 0.02	1.28 ± 0.06	Not measured
**VPR$_{\Delta C}$/I5P**	1.28 ± 0.04	1.16 ± 0.05	1.12 ± 0.07	Not measured
**VPR$_{\Delta C}$/N238P**	1.26 ± 0.07	1.16 ± 0.07	1.09 ± 0.06	Not measured
**VPR$_{\Delta C}$/T265P**	1.25 ± 0.08	1.15 ± 0.08	1.07 ± 0.09	Not measured
**VPR$_{\Delta C}$/N3P/I5P**	1.10 ± 0.04	1.00 ± 0.03	0.94 ± 0.01	11.01 ± 0.27
**VPR$_{\Delta C}$/N3P/I5P/N238P**	1.30 ± 0.10	1.18 ± 0.08	1.13 ± 0.10	Not measured
**VPR$_{\Delta C}$/N3P/I5P/T265P**	1.34 ± 0.01	1.24 ± 0.03	1.17 ± 0.01	Not measured
**VPR$_{\Delta C}$/ N3P/I5P/N238P/T265P**	1.31 ± 0.08	1.19 ± 0.06	1.10 ± 0.05	10.73

Supplementary Table 2. λ_{max} values for proline variants calculated as the maxima of fluorescence curves at different temperatures and for the denatured state at 25 °C. Along with the standard deviation of the mean.

Variant	15 °C (nm)	25 °C (nm)	35 °C (nm)	Denatured 25 °C (nm)
**VPR$_{\Delta C}$**	335 ± 1	335 ± 1	336 ± 1	357 ± 1
**VPR$_{\Delta C}$/N3P**	337 ± 2	337 ± 2	337 ± 2	Not measured
**VPR$_{\Delta C}$/I5P**	337 ± 2	337 ± 2	337 ± 2	Not measured
**VPR$_{\Delta C}$/N238P**	339 ± 1	339 ± 2	339 ± 2	Not measured
**VPR$_{\Delta C}$/T265P**	337 ± 1	338 ± 1	338 ± 1	Not measured
**VPR$_{\Delta C}$/N3P/I5P**	334 ± 1	334 ± 1	334 ± 1	358 ± 1
**VPR$_{\Delta C}$/N3P/I5P/N238P**	339 ± 1	339 ± 1	339 ± 1	Not measured
**VPR$_{\Delta C}$/N3P/I5P/T265P**	338 ± 1	338 ± 1	338 ± 1	Not measured
**VPR$_{\Delta C}$/ N3P/I5P/N238P/T265P**	336 ± 2	336 ± 2	336 ± 2	358

Supplementary Table 3. Stern-Volmer constants calculated from quenching data between 310 nm – 410 nm at different temperatures and for the denatured state at 25 °C. Along with the standard deviation of the mean.

Variant	15 °C (M^{-1})	25 °C (M^{-1})	35 °C (M^{-1})	Denatured 25 °C (M^{-1})
**VPR$_{\Delta C}$**	2.13 ± 0.14	2.24 ± 0.12	2.25 ± 0.26	15.00 ± 0.30
**VPR$_{\Delta C}$/N3P**	2.00 ± 0.12	2.32 ± 0.18	2.66 ± 0.19	Not measured
**VPR$_{\Delta C}$/I5P**	1.96 ± 0.04	2.05 ± 0.13	2.30 ± 0.22	Not measured
**VPR$_{\Delta C}$/N238P**	2.49 ± 0.17	2.63 ± 0.25	2.75 ± 0.23	Not measured
**VPR$_{\Delta C}$/T265P**	2.17 ± 0.08	2.41 ± 0.14	2.45 ± 0.25	Not measured
**VPR$_{\Delta C}$/N3P/I5P**	1.55 ± 0.11	1.64 ± 0.07	1.77 ± 0.02	15.69 ± 0.24
**VPR$_{\Delta C}$/N3P/I5P/N238P**	2.05 ± 0.06	2.18 ± 0.05	2.41 ± 0.23	Not measured
**VPR$_{\Delta C}$/N3P/I5P/T265P**	1.94 ± 0.06	2.17 ± 0.05	2.48 ± 0.07	Not measured
**VPR$_{\Delta C}$/ N3P/I5P/N238P/T265P**	2.05 ± 0.23	2.12 ± 0.19	2.19 ± 0.22	15.67

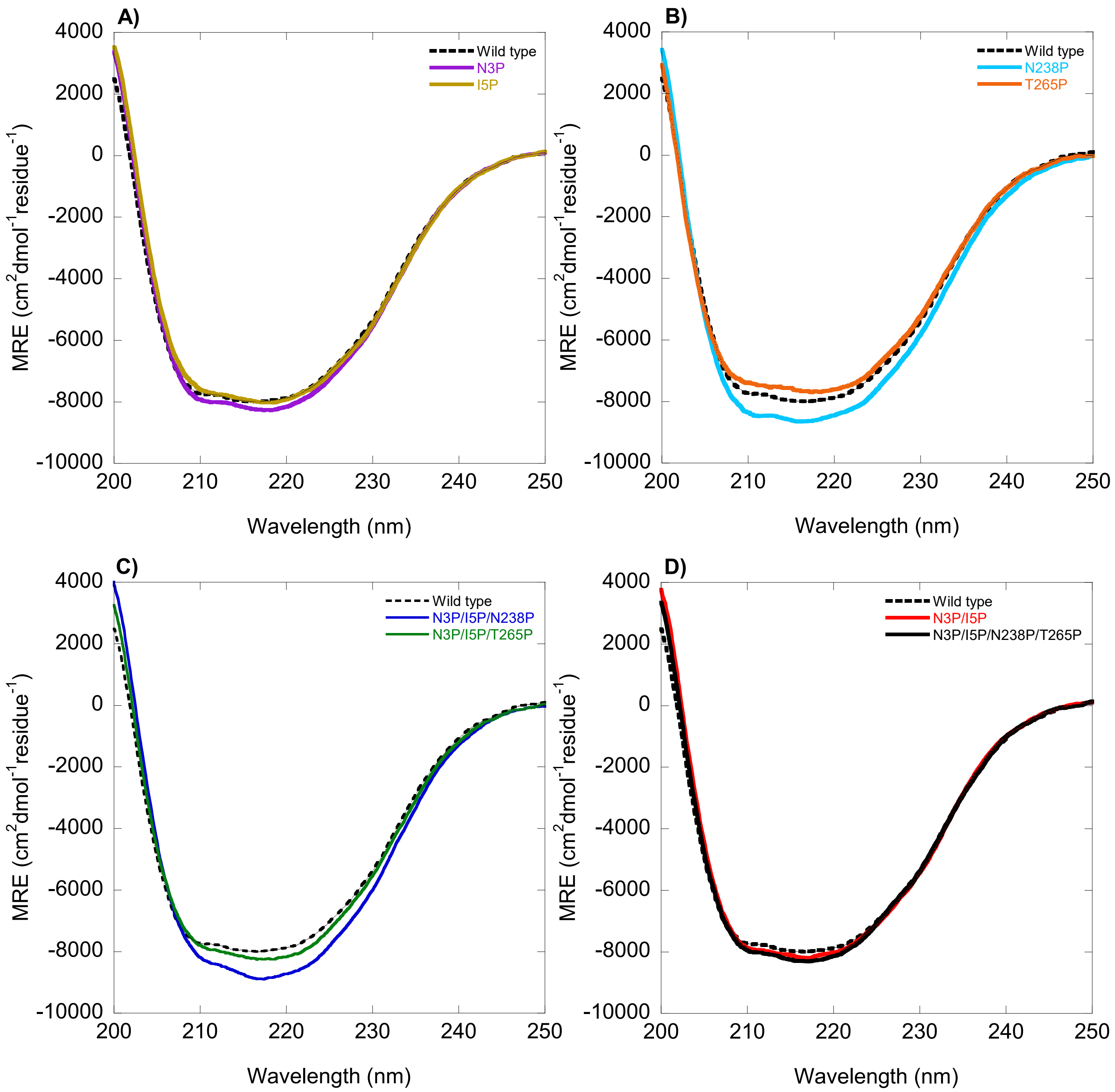

Supplementary Fig. 2. CD wavelength scans of proline variants in glycine buffer containing 15 mM $CaCl_2$ and 100 mM NaCl at 25 °C. $VPR_{\Delta C}$ (black dotted line). **A.** CD wavelength scans of $VPR_{\Delta C}$_N3P (Gold) and $VPR_{\Delta C}$_I5P (purple). **B.** CD wavelength scans of $VPR_{\Delta C}$_N238P (light blue) and $VPR_{\Delta C}$_T265P (orange). **C.** CD wavelength scans of $VPR_{\Delta C}$_N3P/I5P/N238P (blue) and $VPR_{\Delta C}$_N3P/I5P/T265P (green). **D.** CD wavelength scans of $VPR_{\Delta C}$_N3P/I5P (red) and $VPR_{\Delta C}$_N3P/I5P/N238P/T265P (black).

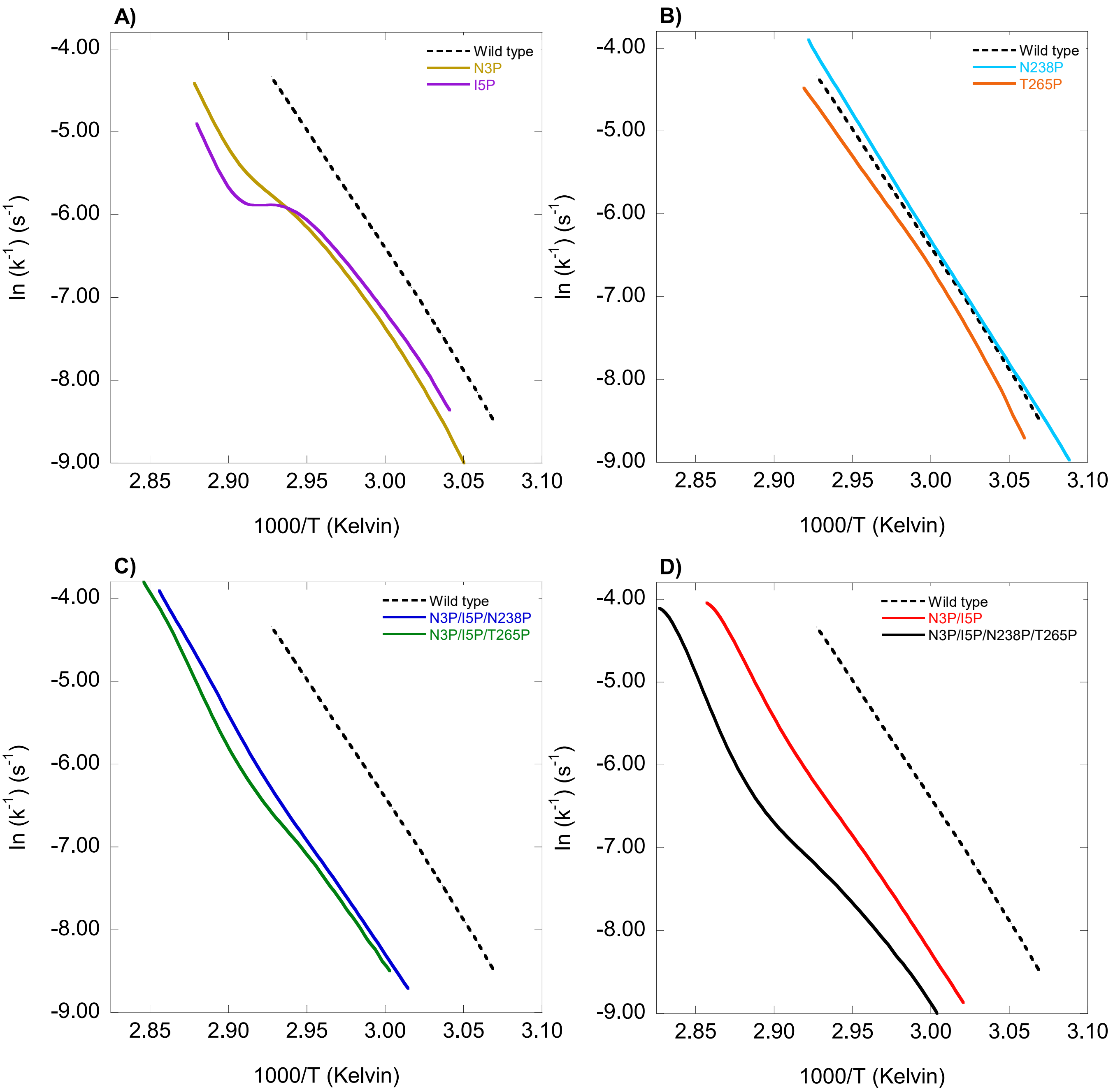

Supplementary Fig. 3. Arrhenius graphs calculated from unfolding rates calculated from DSC thermographs for VPR$_{\Delta C}$ (black dotted line) **A.** VPR$_{\Delta C}$_N3P (Gold) and VPR$_{\Delta C}$_I5P (purple). **B.** VPR$_{\Delta C}$_N238P (light blue) and VPR$_{\Delta C}$_T265P (orange). **C.** VPR$_{\Delta C}$_N3P/I5P/N238P (blue) and VPR$_{\Delta C}$_N3P/I5P/T265P (green). **D.** VPR$_{\Delta C}$_N3P/I5P (red) and VPR$_{\Delta C}$_N3P/I5P/N238P/T265P (black).

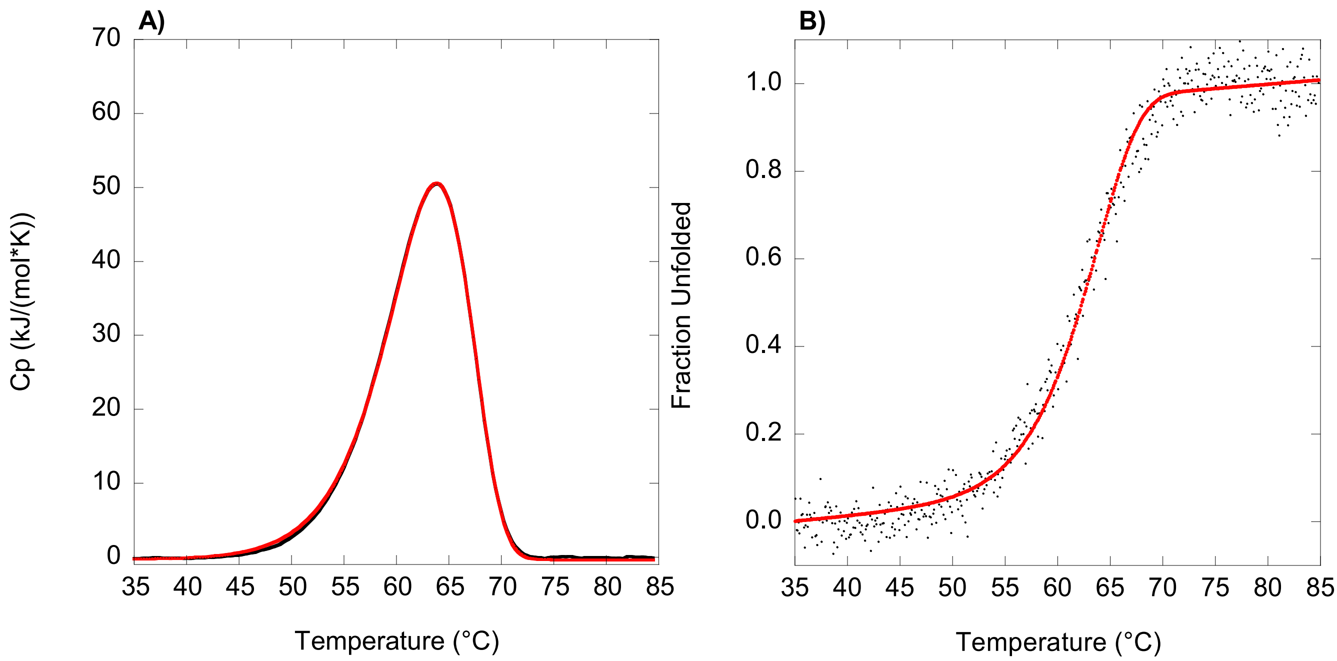

Supplementary Fig. 4. CalFitter 1.2 global fits (red line) of $VPR_{\Delta C}$ normalized DSC thermogram **(A)** (black line) and CD melting profile **(B)** (black dots). Assay conditions: 25 mM glycine, 15 mM $CaCl_2$ and 100 mM NaCl at pH 8.6. Global fit parameters were: E_{act} = 235 ± 2 kJ/mol, T_{act} = 87.4 ± 0.3 °C and $\Delta H_{cal\text{-}fit}$ = 542 ± 5 kJ/mol.

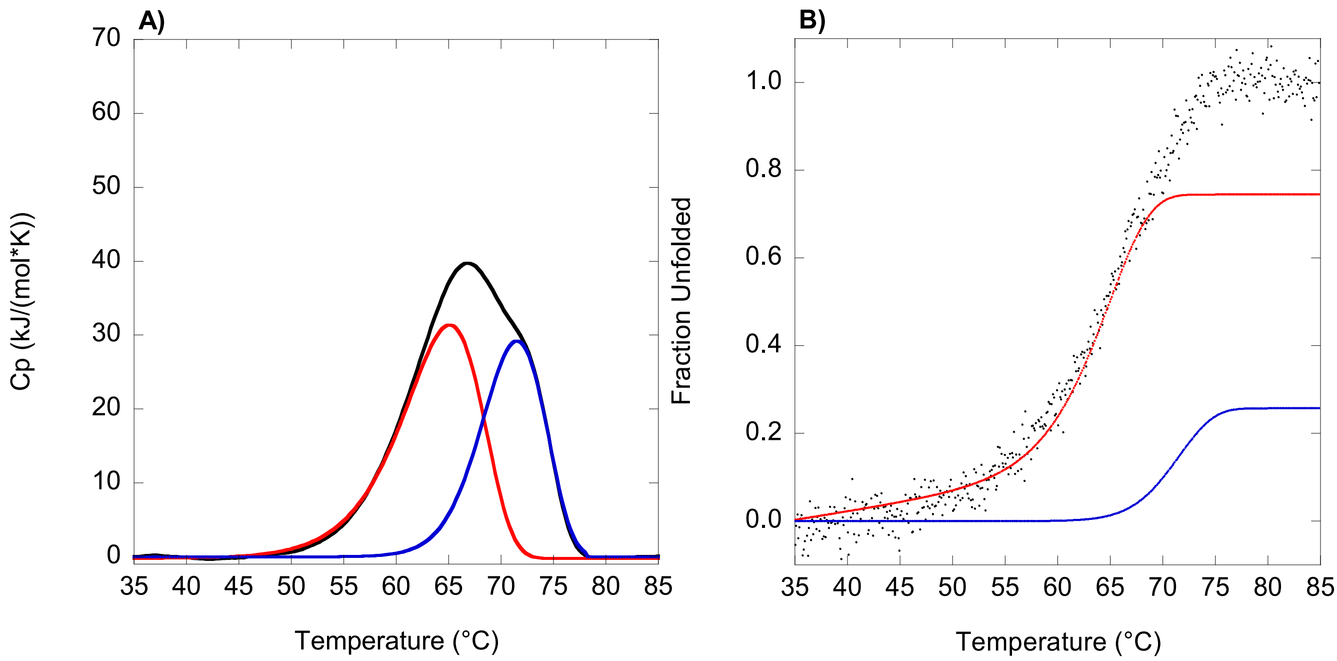

Supplementary Fig. 5. CalFitter 1.2 global fits (red line for transition 1 and blue line for transition 2) of $VPR_{\Delta C}$_N3P normalized DSC thermogram **(A)** (black line) and CD melting profile **(B)** (black dots). Assay conditions: 25 mM glycine, 15 mM $CaCl_2$ and 100 mM NaCl at pH 8.6. Global fit parameters were: E_{act}^1 = 251 ± 5 kJ/mol, E_{act}^2 = 285 ± 20 kJ/mol, T_{act}^1 = 87.0 ± 0.8 °C, T_{act}^2 = 90.8 ± 1.3 °C, $\Delta H_{cal\text{-}fit}^1$ = 318 ± 20 kJ/mol and $\Delta H_{cal\text{-}fit}^2$ = 227 ± 19 kJ/mol.

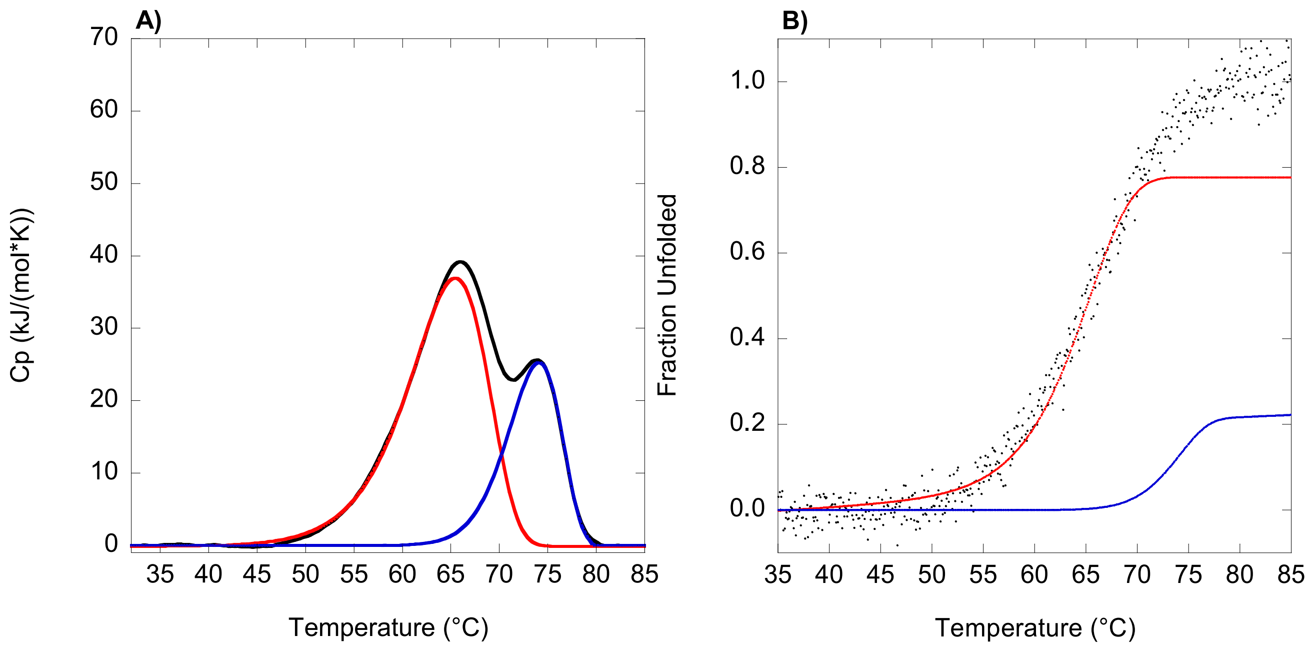

Supplementary Fig. 6. CalFitter 1.2 global fits (red line for transition 1 and blue line for transition 2) of $VPR_{\Delta C}$_I5P normalized DSC thermogram **(A)** (black line) and CD melting profile **(B)** (black dots). Assay conditions: 25 mM glycine, 15 mM $CaCl_2$ and 100 mM NaCl at pH 8.6. Global fit parameters were: E_{act}^1 = 235 ± 3 kJ/mol, E_{act}^2 = 356 ± 13 kJ/mol, T_{act}^1 = 89.3 ± 0.4 °C, T_{act}^2 = 89.2 ± 0.6 °C, $\Delta H_{cal\text{-}fit}^1$ = 399 ± 7 kJ/mol and $\Delta H_{cal\text{-}fit}^2$ = 180 ± 6 kJ/mol.

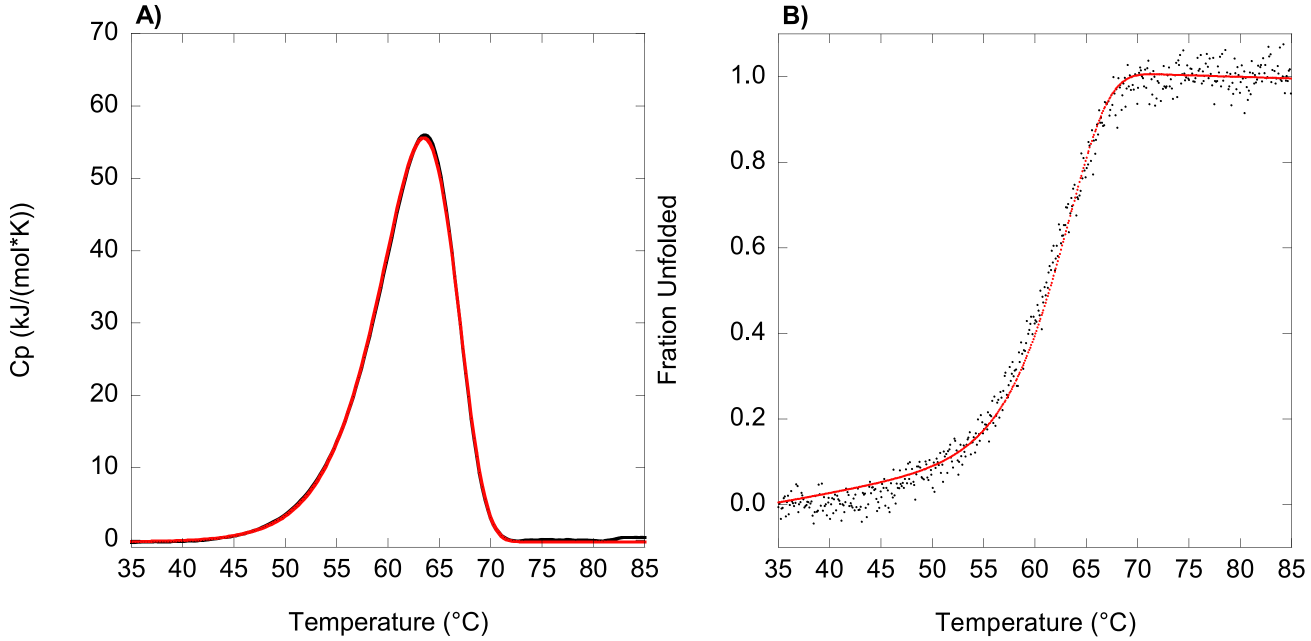

Supplementary Fig. 7. CalFitter 1.2 global fits (red line) of $VPR_{\Delta C}$_N238P normalized DSC thermogram **(A)** (black line) and CD melting profile **(B)** (black dots). Assay conditions: 25 mM glycine, 15 mM $CaCl_2$ and 100 mM NaCl at pH 8.6. Global fit parameters were: E_{act} = 248 ± 2 kJ/mol, T_{act} = 85.5 ± 0.2 °C and $\Delta H_{cal\text{-}fit}$ = 564 ± 4 kJ/mol.

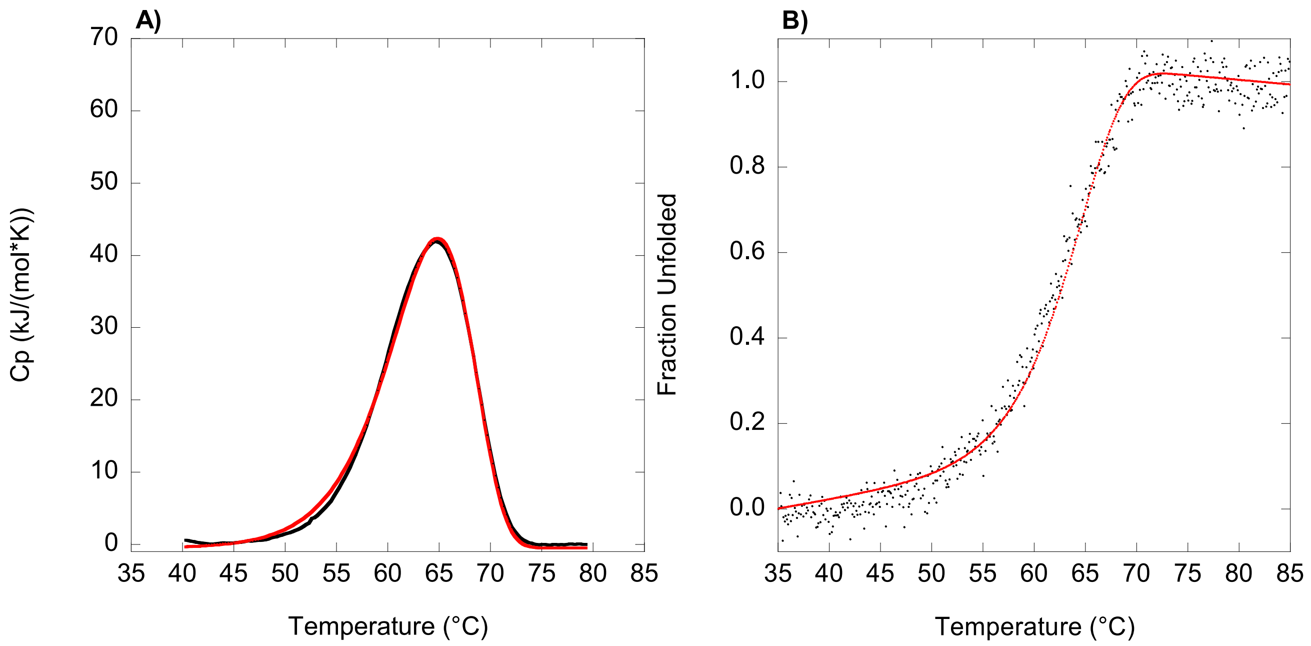

Supplementary Fig. 8. CalFitter 1.2 global fits (red line) of $VPR_{\Delta C}$_T265P normalized DSC thermogram **(A)** (black line) and CD melting profile **(B)** (black dots). Assay conditions: 25 mM glycine, 15 mM $CaCl_2$ and 100 mM NaCl at pH 8.6. Global fit parameters were: E_{act} = 229 ± 2 kJ/mol, T_{act} = 89.4 ± 0.3 °C and $\Delta H_{cal\text{-}fit}$ = 471 ± 5 kJ/mol.

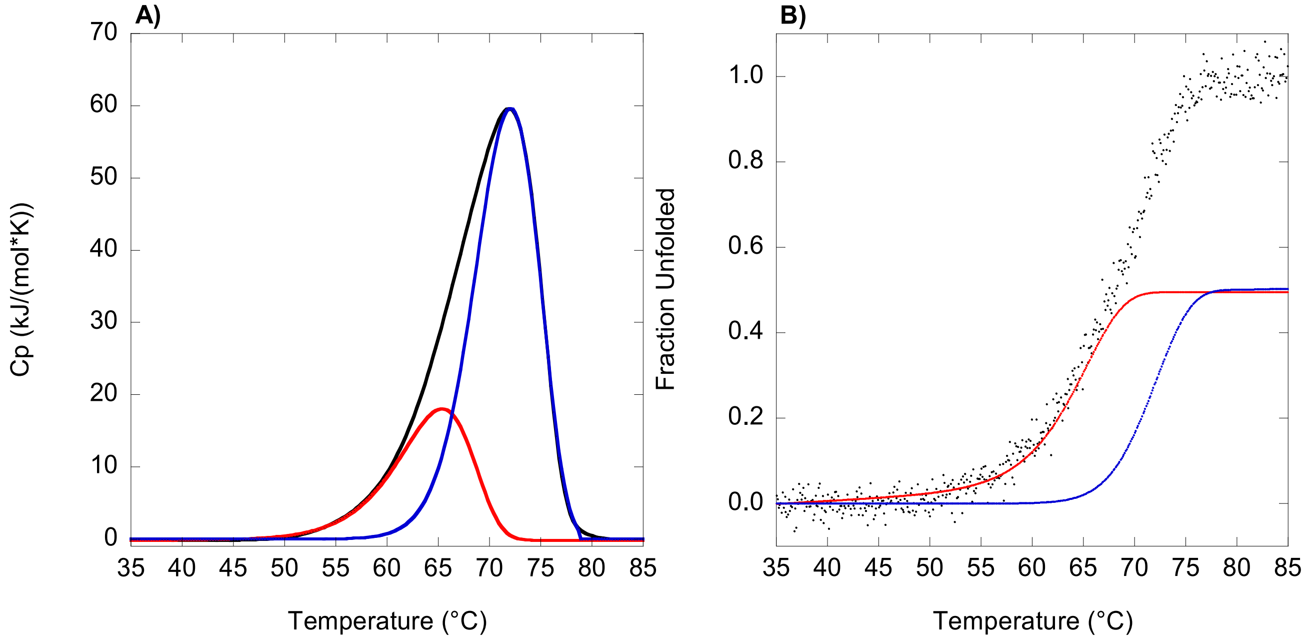

Supplementary Fig. 9. CalFitter 1.2 global fits (red line for transition 1 and blue line for transition 2) of $VPR_{\Delta C}$_N3P/I5P normalized DSC thermogram **(A)** (black line) and CD melting profile **(B)** (black dots). Assay conditions: 25 mM glycine, 15 mM $CaCl_2$ and 100 mM NaCl at pH 8.6. Global fit parameters were: E_{act}^{1} = 261 ± 9 kJ/mol, E_{act}^{2} = 283 ± 6 kJ/mol, T_{act}^{1} = 86.2 ± 1.3 °C, T_{act}^{2} = 91.8 ± 0.4 °C, $\Delta H_{cal\text{-}fit}^{1}$ = 176 ± 20 kJ/mol and $\Delta H_{cal\text{-}fit}^{2}$ = 477 ± 19 kJ/mol.

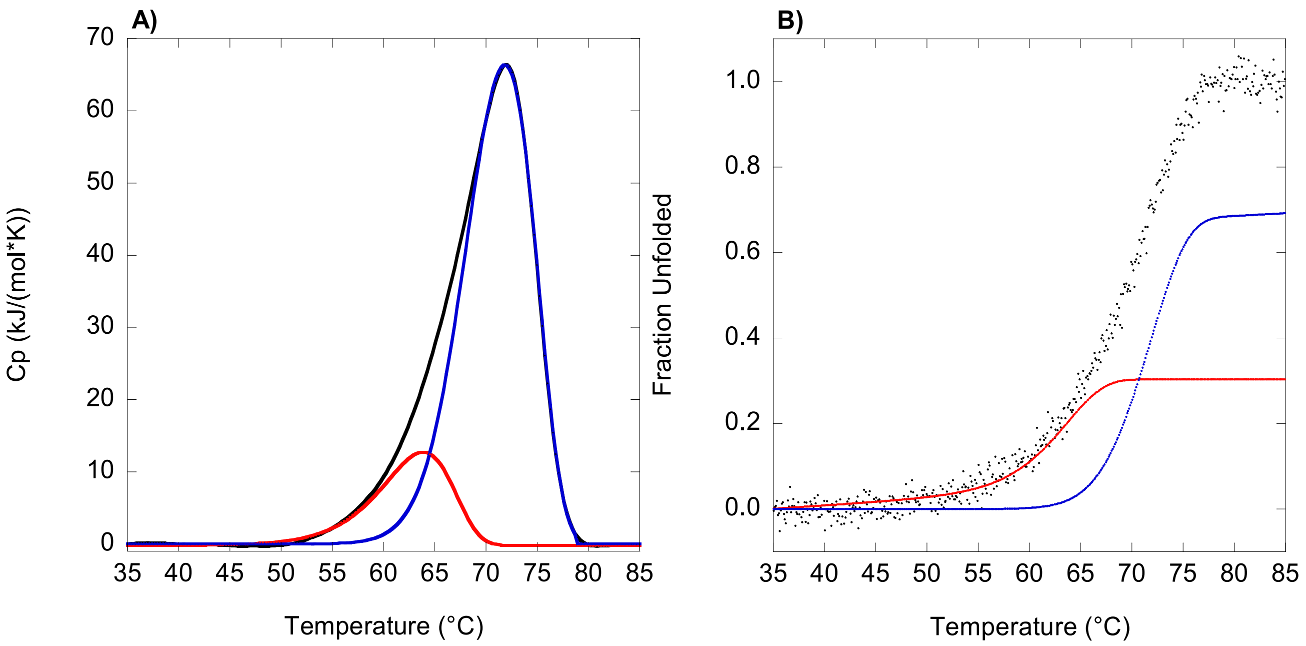

Supplementary Fig. 10. CalFitter 1.2 global fits (red line for transition 1 and blue line for transition 2) of $VPR_{\Delta C}$_N3P/I5P/N238P normalized DSC thermogram **(A)** (black line) and CD melting profile **(B)** (black dots). Assay conditions: 25 mM glycine, 15 mM $CaCl_2$ and 100 mM NaCl at pH 8.6. Global fit parameters were: E_{act}^1 = 270 ± 11 kJ/mol, E_{act}^2 = 279 ± 3 kJ/mol, T_{act}^1 = 83.6 ± 1.4 °C, T_{act}^2 = 92.0 ± 0.3 °C, $\Delta H_{cal\text{-}fit}^1$ = 120 ± 13 kJ/mol and $\Delta H_{cal\text{-}fit}^2$ = 567 ± 13 kJ/mol.

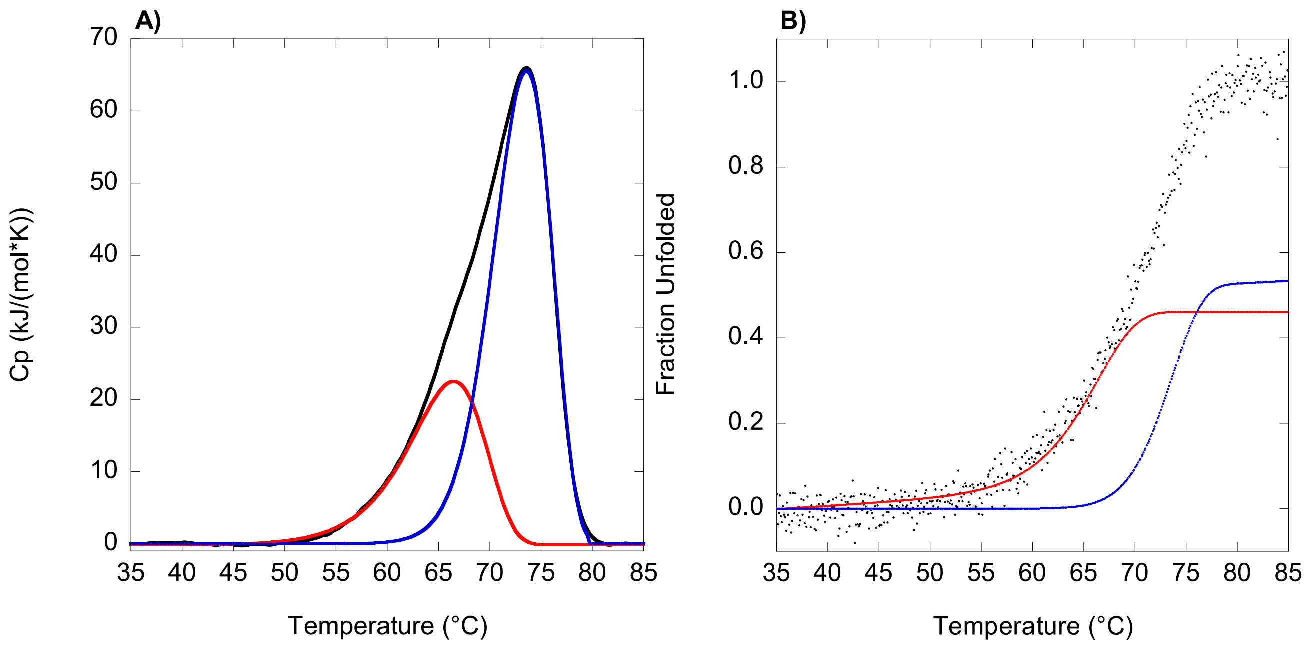

Supplementary Fig. 11. CalFitter 1.2 global fits (red line for transition 1 and blue line for transition 2) of $VPR_{\Delta C}$_N3P/I5P/T265P normalized DSC thermogram **(A)** (black line) and CD melting profile **(B)** (black dots). Assay conditions: 25 mM glycine, 15 mM $CaCl_2$ and 100 mM NaCl at pH 8.6. Global fit parameters were: E_{act}^1 = 259 ± 10 kJ/mol, E_{act}^2 = 326 ± 8 kJ/mol, T_{act}^1 = 87.7 ± 1.4 °C, T_{act}^2 = 90.3 ± 0.4 °C, $\Delta H_{cal\text{-}fit}^1$ = 224 ± 20 kJ/mol and $\Delta H_{cal\text{-}fit}^2$ = 481 ± 18 kJ/mol.

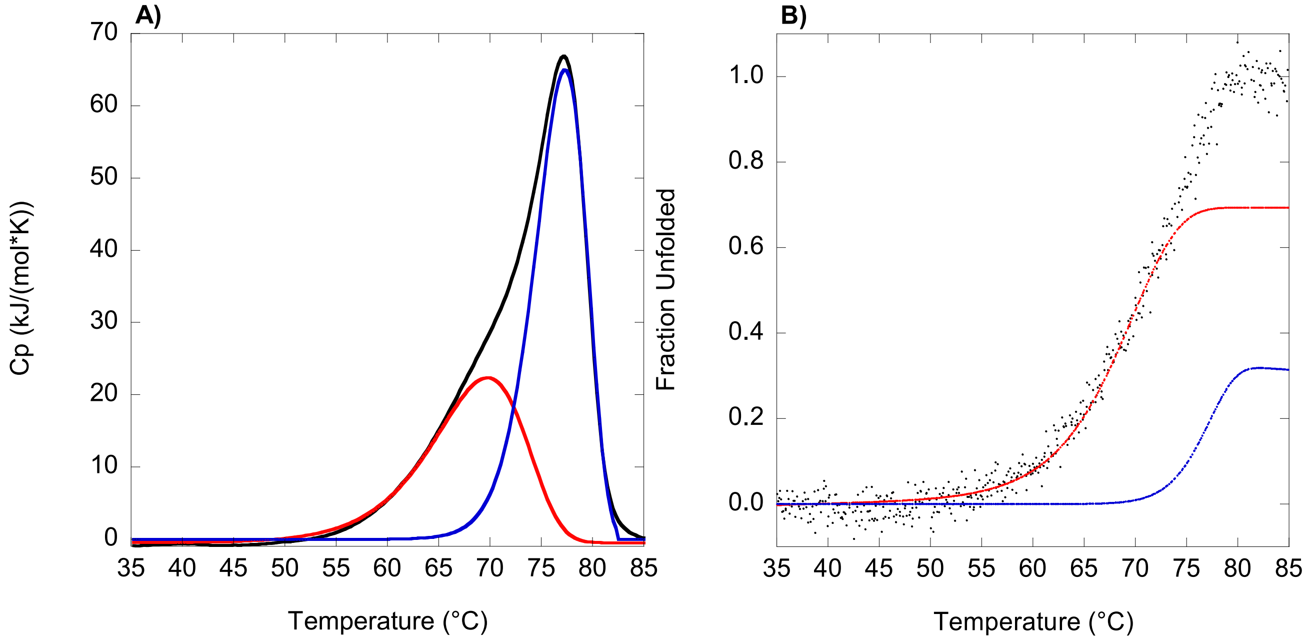

Supplementary Fig. 12. CalFitter 1.2 global fits (red line for transition 1 and blue line for transition 2) of $VPR_{\Delta C}$_N3P/I5P/N238P/T265P normalized DSC thermogram **(A)** (black line) and CD melting profile **(B)** (black dots). Assay conditions: 25 mM glycine, 15 mM $CaCl_2$ and 100 mM NaCl at pH 8.6. Global fit parameters were: E_{act}^{1} = 215 ± 8 kJ/mol, E_{act}^{2} = 383 ± 7 kJ/mol, T_{act}^{1} = 97.3 ± 1.6 °C, T_{act}^{2} = 91.3 ± 0.3 °C, $\Delta H_{cal\text{-}fit}^{1}$ = 275 ± 17 kJ/mol and $\Delta H_{cal\text{-}fit}^{2}$ = 425 ± 15 kJ/mol.

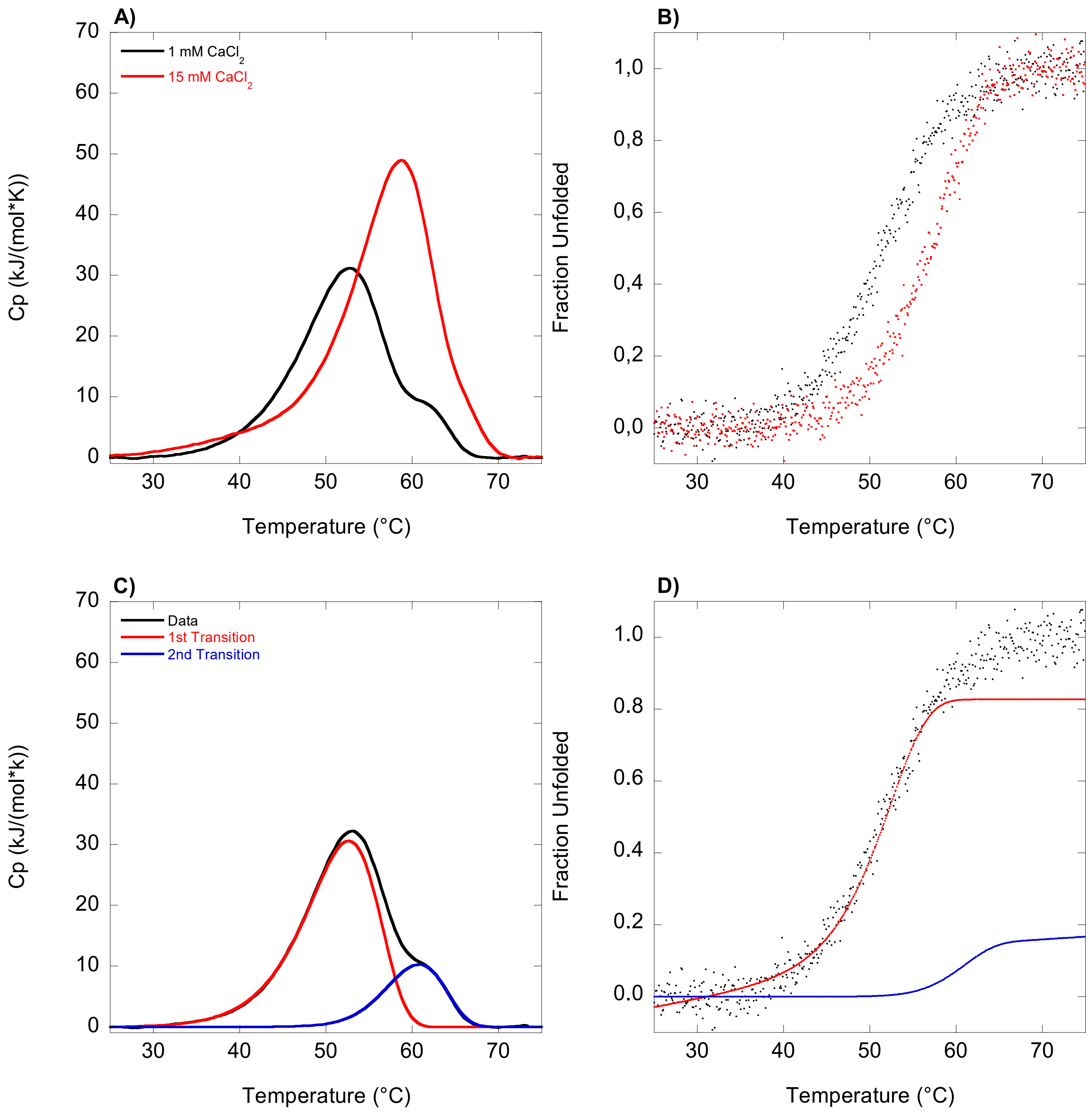

Supplementary Fig. 13. DCS thermogram **(A)** and CD melting profile **(B)** of VPR$_{\Delta C}$ at two different calcium ion concentrations, 1 mM (black line) and 15 mM (red line) at pH 5.0 in a 25 mM acetate buffer and 100 mM NaCl. CalFitter 1.2 global fits (red line for transition 1 and blue line for transition 2) of VPR$_{\Delta C}$ at 1 mM $CaCl_2$ and pH 5.0 of normalized DSC thermogram (black line) **(C)** and normalized CD melting profile (black dots) **(D)** Global fit parameters were: E_{act}^1 = 210 ± 3 kJ/mol, E_{act}^2 = 252 ± 25 kJ/mol, T_{act}^1 = 77.7 ± 0.5 °C, T_{act}^2 = 81.9 ± 2.2 °C, $\Delta H_{cal\text{-}fit}^1$ = 342 ± 9 kJ/mol and $\Delta H_{cal\text{-}fit}^2$ = 89 ± 8 kJ/mol.

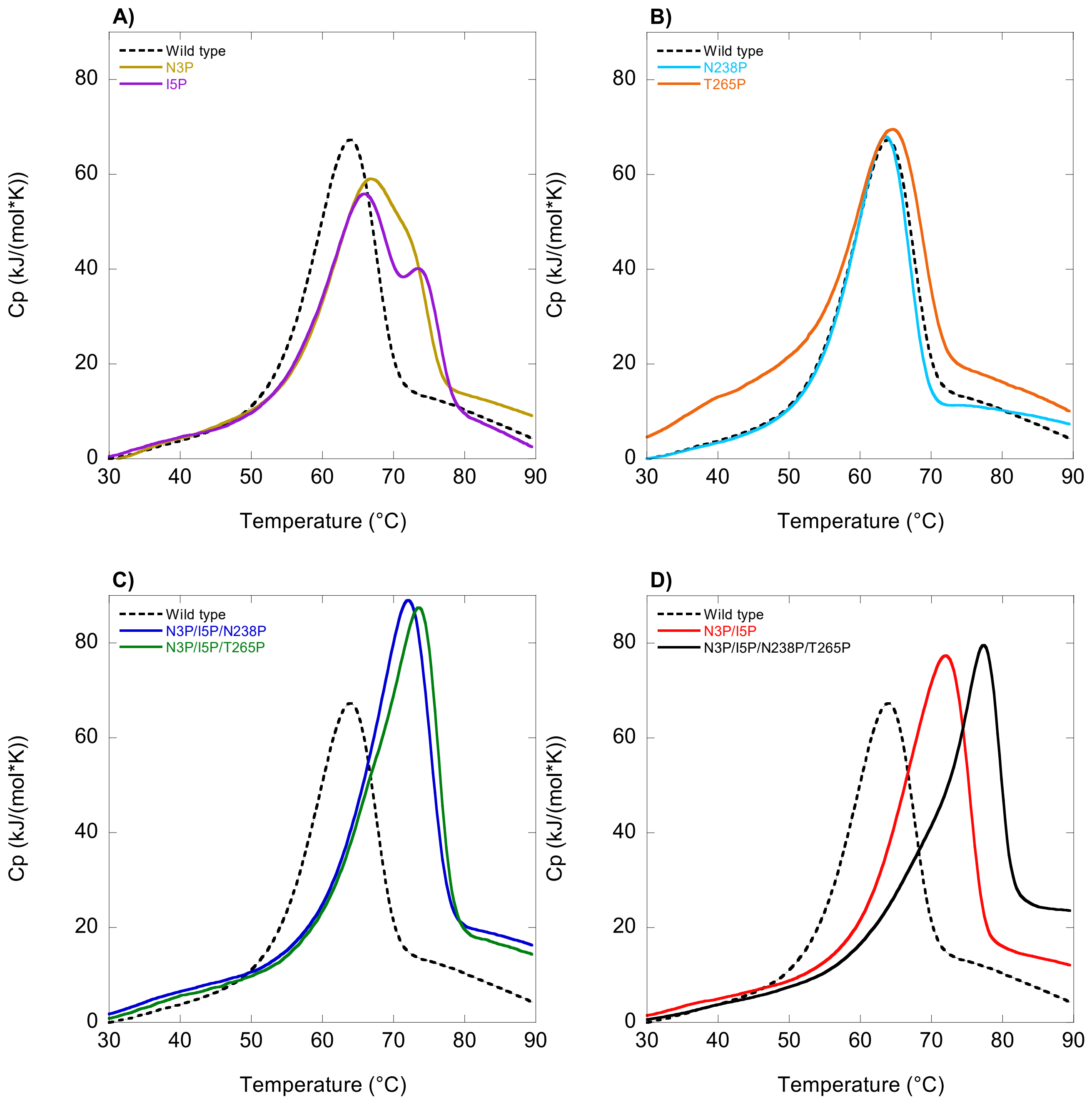

Supplementary Fig. 14. Buffer subtracted differential scanning thermograms showing the excess heat during the unfolding process of the proline variants in a 25 mM glycine buffer containing 15 mM $CaCl_2$ and 100 mM NaCl. $VPR_{\Delta C}$ (dotted black line). **(A)** Unfolding of $VPR_{\Delta C}$_N3P (Gold) and $VPR_{\Delta C}$_I5P (purple). **(B)** Unfolding of $VPR_{\Delta C}$_N238P (light blue) and $VPR_{\Delta C}$_T265P (orange). **(C)** Unfolding of $VPR_{\Delta C}$_N3P/I5P/N238P (blue) and $VPR_{\Delta C}$_N3P/I5P/T265P (green). **(D)** Unfolding of $VPR_{\Delta C}$_N3P/I5P (red) and $VPR_{\Delta C}$_N3P/I5P/N238P/T265P (black).

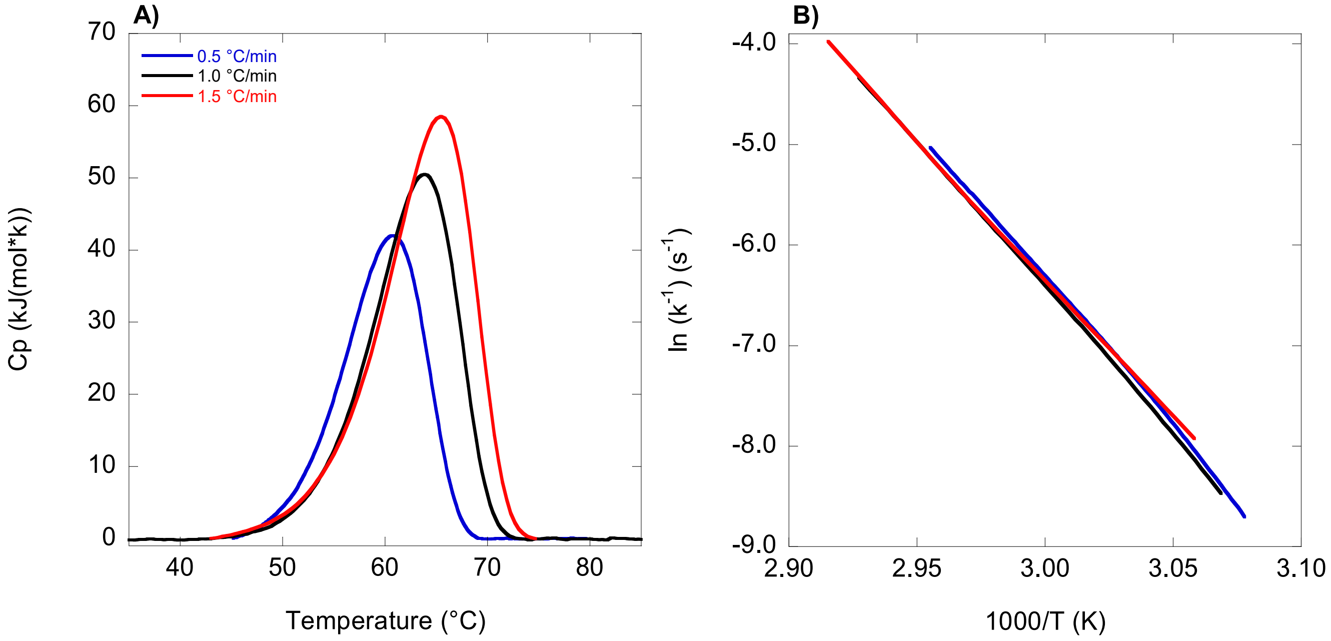

Supplementary Fig. 15. (A) Thermograms of $VPR_{\Delta C}$ in a 25 mM glycine buffer containing 15 mM $CaCl_2$ and100 mM NaCl at three different scan rates, 0.5 °C/min (blue line), 1.0 °C/min (black line) and 1.5 °C/min (red line). **(B)** Arrhenius graph showing the rate of unfolding at three different scan rates calculated from $VPR_{\Delta C}$ thermograms 25 mM glycine buffer containing 15 mM $CaCl_2$ and100 mM NaCl, 0.5 °C/min (blue line), 1.0 °C/min (black line) and 1.5 °C/min (red line).

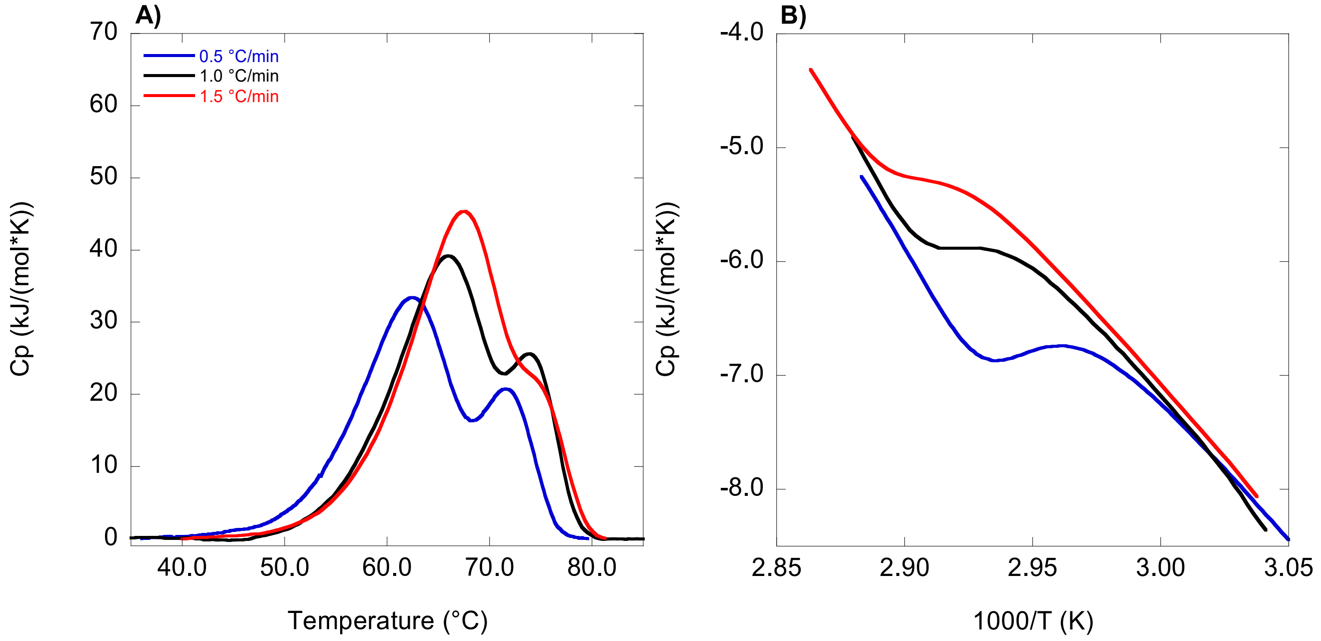

Supplementary Fig. 16. (A) Thermograms of $VPR_{\Delta C}$_I5P in a 25 mM glycine buffer containing 15 mM $CaCl_2$ and100 mM NaCl at three different scan rates, 0.5 °C/min (blue line), 1.0 °C/min (black line) and 1.5 °C/min (red line). **(B)** Arrhenius graph showing the rate of unfolding at three different scan rates calculated from $VPR_{\Delta C}$_I5P thermograms 25 mM glycine buffer containing 15 mM $CaCl_2$ and100 mM NaCl, 0.5 °C/min (blue line), 1.0 °C/min (black line) and 1.5 °C/min (red line).

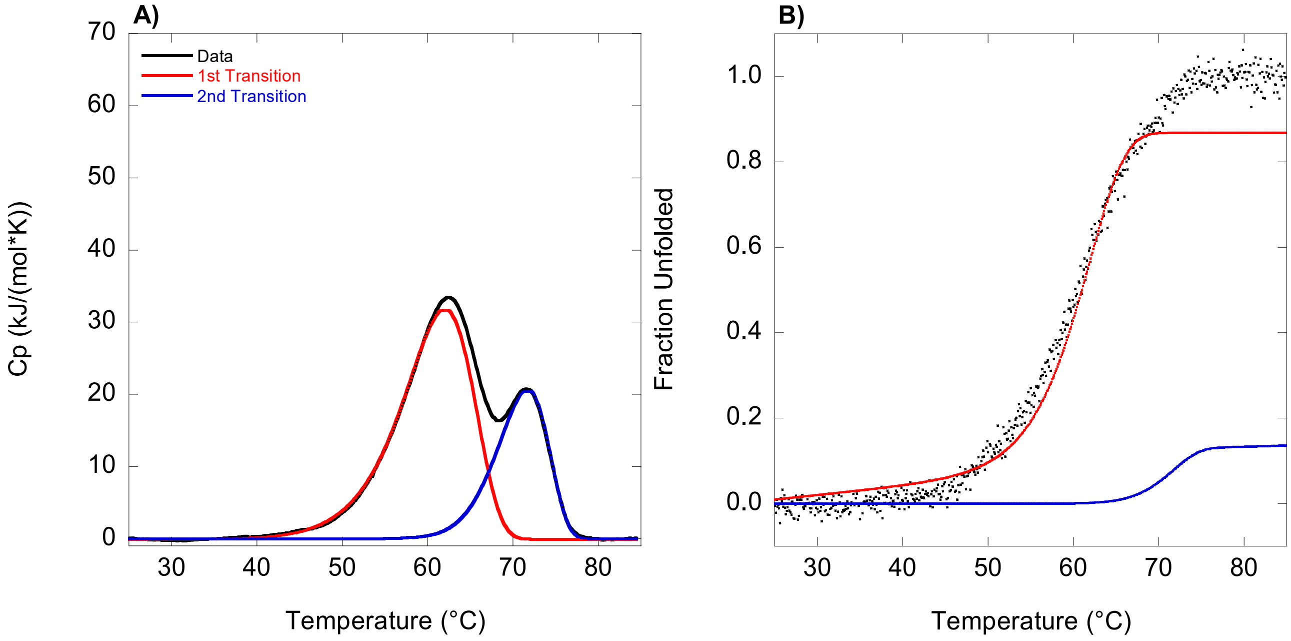

Supplementary Fig. 17. CalFitter 1.2 global fits (red line for transition 1 and blue line for transition 2) of $VPR_{\Delta C}$_I5P normalized DSC thermogram **(A)** (black line) and CD melting profile **(B)** (black dots). Assay conditions: 0.5 °C/min, 25 mM glycine, 15 mM $CaCl_2$ and 100 mM NaCl at pH 8.6. Global fit parameters were: E_{act}^1 = 227 ± 2 kJ/mol, E_{act}^2 = 330 ± 7 kJ/mol, T_{act}^1 = 89.6 ± 0.3 °C, T_{act}^2 = 90.3 ± 0.5 °C, $\Delta H_{cal\text{-}fit}^1$ = 348 ± 3 kJ/mol and $\Delta H_{cal\text{-}fit}^2$ = 158 ± 3 kJ/mol.

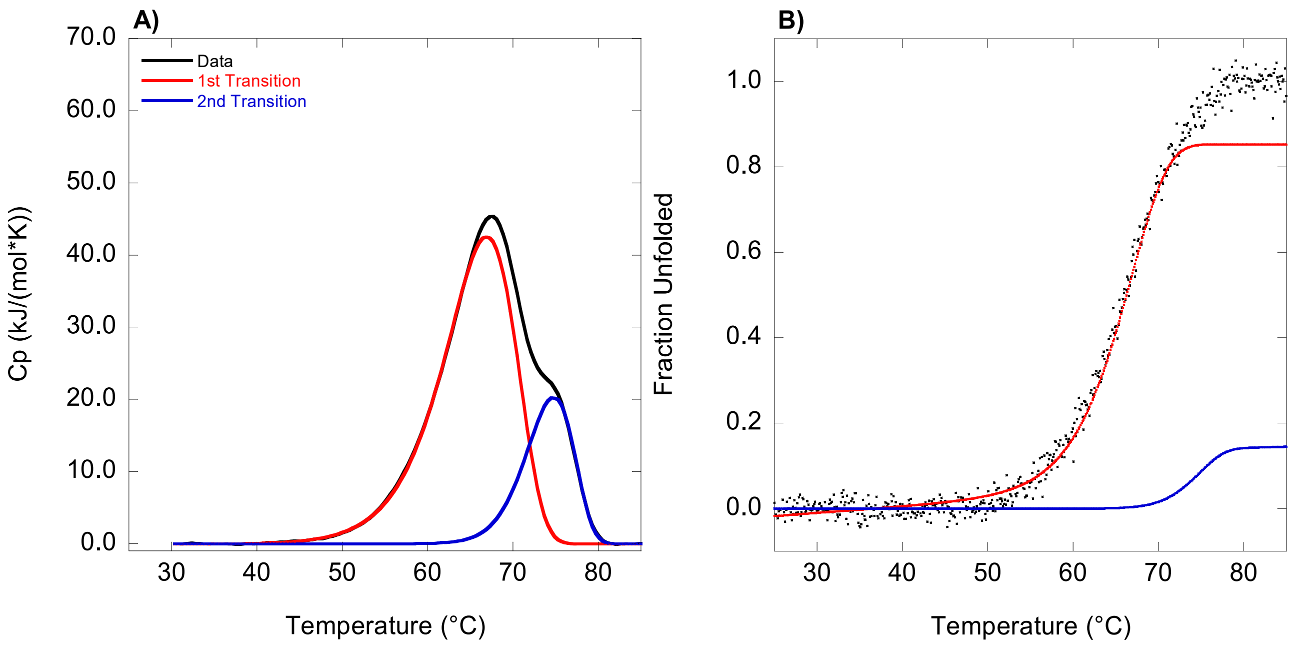

Supplementary Fig. 18. CalFitter 1.2 global fits (red line for transition 1 and blue line for transition 2) of VPR$_{\Delta C}$_I5P normalized DSC thermogram **(A)** (black line) and CD melting profile **(B)** (black dots). Assay conditions: 1.5 °C/min, 25 mM glycine, 15 mM $CaCl_2$ and 100 mM NaCl at pH 8.6. Global fit parameters were: E_{act}^1 = 229 ± 2 kJ/mol, E_{act}^2 = 338 ± 16 kJ/mol, T_{act}^1 = 89.9 ± 0.4 °C, T_{act}^2 = 89.5 ± 0.7 °C, $\Delta H_{cal\text{-}fit}^1$ = 475 ± 8 kJ/mol and $\Delta H_{cal\text{-}fit}^2$ = 147 ± 7 kJ/mol.

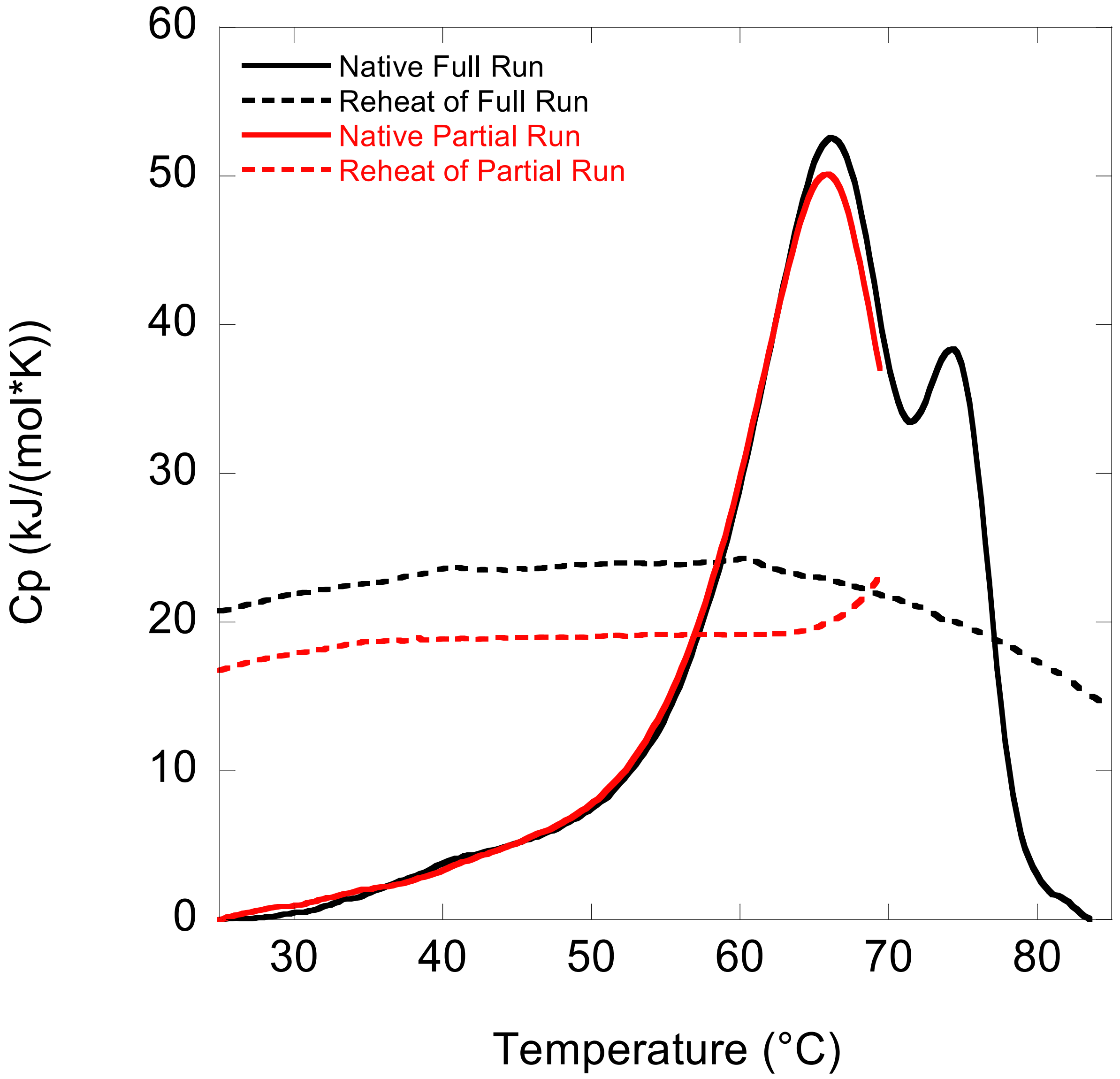

Supplementary Fig. 19. Complete (black line) and partial (red line) unfolding of $VPR_{\Delta C}$_I5P followed by a reheating of complete unfolding (black dotted line) and reheating of partial unfolding (red dotted line). Assay conditions: 1.0 °C/min, 25 mM glycine, 15 mM $CaCl_2$ and 100 mM NaCl at pH 8.6.

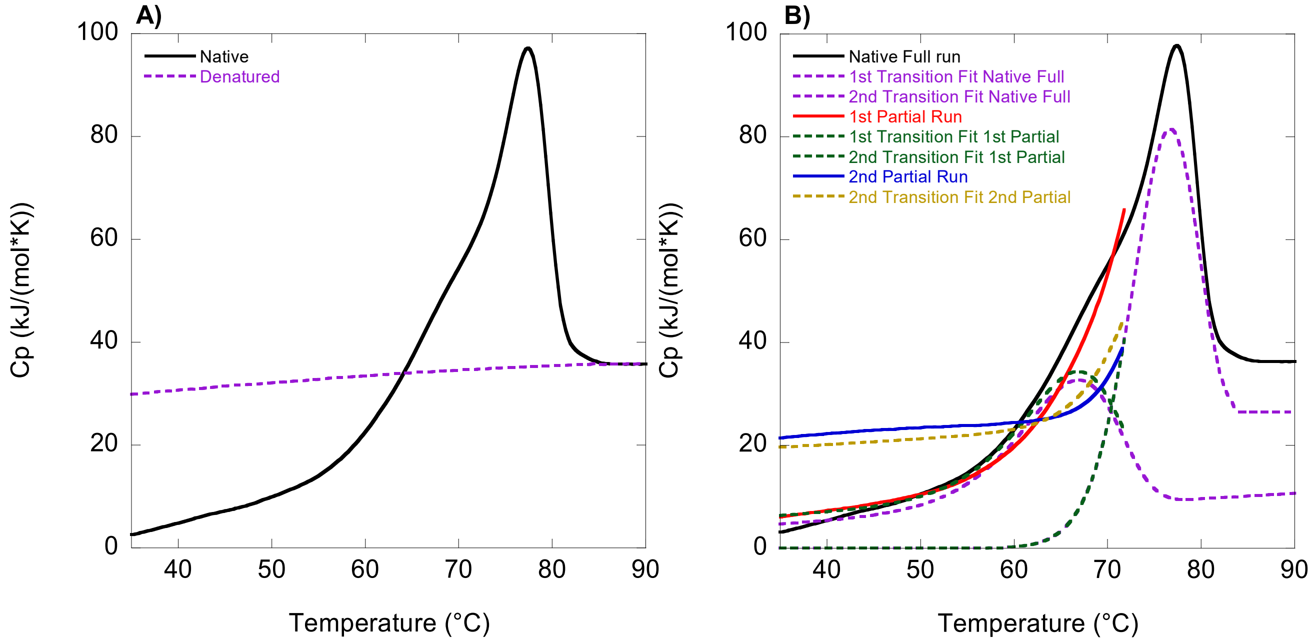

Supplementary Fig. 20. A) Complete unfolding of VPR$_{\Delta C}$_N3P/I5P/N238P/T265P (black line) followed by reheating of the resulting denatured assembly (purple dotted line) confirming the irreversibility of the full unfolding process. **B)** Partial unfolding of VPR$_{\Delta C}$_N3P/I5P/N238P/T265P fitted using CalFitter 1.2. CalFitter analysis was carried out using the reheat feature in the program. Input data consisted of three thermograms: total denaturation of the protein (black solid line and fitted curves purple dotted lines), partial unfolding of the protein by heating a native sample to 72 °C (red solid line and fitted curves green dotted lines) and reheating of that partially unfolded sample to 72 °C (blue solid line and the fitted curve golden dotted line). Prior to fitting via CalFitter samples were subjected to thermocycle baseline subtraction. The best fitting model was a three-state model with two irreversible transitions. Assay conditions were 25 mM glycine, 15 mM $CaCl_2$ and 100 mM NaCl at pH 8.6.

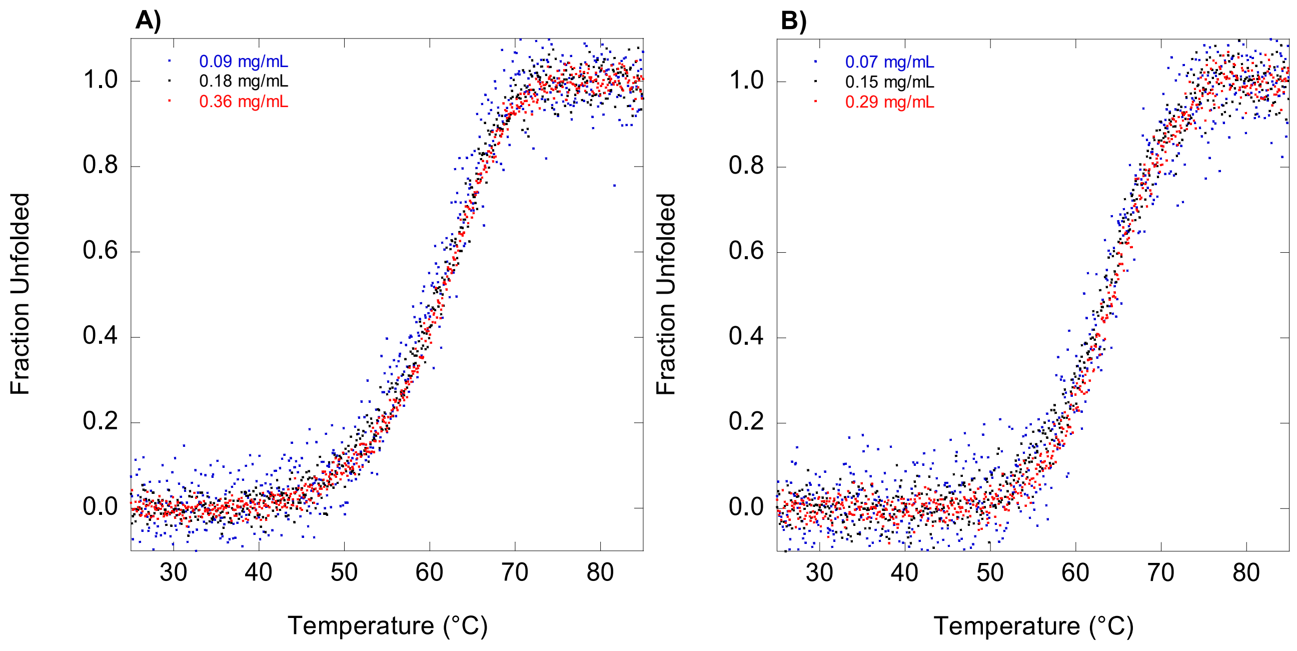

Supplementary Fig. 21. Protein concentration effects on melting points measured on CD of $VPR_{\Delta C}$ **(A)** and $VPR_{\Delta C}$_I5P **(B)**. The three protein concentrations tested were approximately 0.1 mg/mL (blue dots), 0.2 mg/mL (black dots) and 0.4 mg/mL (red dots). Assay conditions were 25 mM glycine, 15 mM $CaCl_2$ and 100 mM NaCl at pH 8.6.

References.

1. Mazurenko, S. *et al.* CalFitter: a web server for analysis of protein thermal denaturation data. *Nucleic Acids Res* **46**, W344-W349, doi:10.1093/nar/gky358 (2018).
2. Jaswal, S. S., Truhlar, S. M. E., Dill, K. A. & Agard, D. A. Comprehensive analysis of protein folding activation thermodynamics reveals a universal behavior violated by kinetically stable proteases. *J. Mol. Biol.* **347**, 355-366, doi:10.1016/j.jmb.2005.01.032 (2005).
